# Self-Referential Generalized-Cylinder Fitting for Mast Deformation Measurement from UAV Point Clouds

**DOI:** 10.3390/s26216938

**Published:** 2026-11-02

**Authors:** Ana Kuzmanic Skelin, Mirjana Bonkovic, Mario Milicevic, Mojmil Cecic

**Affiliations:** 1 Faculty of Electrical Engineering, Mechanical Engineering and Naval Architecture (FESB), University of Split, 21000 Split, Croatia; mirjana@fesb.hr (M.B.); mcecic@fesb.hr (M.C.); 2 Faculty of Maritime Studies, University of Split, 21000 Split, Croatia; mmilicevi@pfst.hr

**Keywords:** UAV photogrammetry, structure from motion, point cloud, generalized cylinder, photogrammetric doming, terrestrial laser scanning, structural deformation, dimensional control, ship mast

## Abstract

Dimensional control of tall-ship masts is difficult: rigging occludes deck-based surveys, and registration to a design model assumes it matches the as-built structure. We present a deterministic, self-referential method that measures mast bending from an unmanned aerial vehicle (UAV) photogrammetric point cloud without a computer-aided design model. Each mast is modeled as a tapered generalized cylinder: slices are fitted with trimmed circles, flare and rigging-band slices are rejected, and an uncertainty-weighted centerline is split into rake and bending. A five-mast shared-curvature test provides a candidate bound on photogrammetric doming but is inconclusive here. On the *Golden Horizon*, the method recovers aft rakes of 1.2–1.9° and bends of 36–160 mm at wavelengths above 16 m. On matched synthetic clouds, assumed spatially correlated reconstruction error sets per-mast floors of 20–27 mm (mean) and 29–40 mm (90th percentile), against 7 mm under independent noise; UAV and terrestrial laser scanning (TLS) profiles have root-mean-square differences of 14–33 mm. Tested against straight synthetic masts, two masts bend significantly at the 5% level, but only the Foremast, which TLS could not check, stays significant under all fourteen error models and, under the default model, after a multiple-testing correction; the Mainmast, significant under eleven, agrees with TLS only transversely.

## 1. Introduction

Large sail-powered vessels bring a specific dimensional-control requirement: the geometry of their very tall masts must be verified during construction as part of quality control and in service as part of structural-health monitoring. Such masts are slender steel tubes tens of meters high, assembled, aligned, and tensioned against extensive standing rigging; small deviations from the intended geometry can be visible to the eye yet difficult to quantify. The vessel studied here, the *Golden Horizon*, is a five-masted barque built at the Brodosplit shipyard in Split, Croatia, and is recognized as the world’s largest sailing ship [1]. Its design is based on that of the French barque *France II*, launched in 1911. The masts rise approximately 42–54 m above the deck, and each is a tapered steel tube raked aft in accordance with the vessel’s design. In addition to self-weight, the masts are loaded by the yards of the four square-rigged masts, by the standing rigging and by operational environmental forces. Verifying the deviation of these masts from their intended geometry is therefore a demanding dimensional-control problem.

Conventional survey methods struggle with this geometry. Deck-based total stations and terrestrial laser scanning (TLS) are limited by line of sight in a dense forest of shrouds, stays, and yards and by the difficulty of reaching the upper and inter-mast regions from the deck. In the present dataset, one mast is covered by fewer than 2000 TLS points, which is insufficient for a reliable fit (Section 4.8). Measurements referenced to gravity or to the shore are, in addition, disturbed by the motion of the floating hull: a change in tilt of only 0.1° during a survey displaces the top of a 50 m mast by about 90 mm, which is comparable to the bends to be measured. An earlier reverse-engineering campaign on this vessel reconstructed the masts by laser scanning, followed by manual alignment to the as-designed computer-aided design (CAD) model and circle-loft modeling in CAD software [2]. That approach is labor-intensive, requires a trusted design model, and, most importantly, makes the measured deformation depend on a manual alignment step whose error propagates into every result. The limitation lies in the alignment rather than in the scanning: the TLS data of that campaign are reprocessed here with the proposed pipeline and serve as an independent reference (Section 3.1). What is needed is a model-free, automated measurement that refers each mast to its own axis so that the result depends neither on a design model nor on the attitude of the hull.

Unmanned aerial vehicle (UAV) photogrammetry is an attractive alternative. A UAV can view the masts from angles unreachable from the deck, including the occluded upper sections, and structure-from-motion (SfM) reconstruction yields a dense three-dimensional (3D) point cloud at low operational cost [3,4]. The same richness, however, brings difficulties. The cloud is cluttered by rigging and attachments; a mast is a tapered tube whose axis is itself a curve when deformed, so fitting a single cylinder confounds taper with bending; and, as a hazard specific to photogrammetry, SfM reconstruction can contain a broad-scale systematic curvature (doming) that bends a perfectly straight object and is indistinguishable from real deformation unless explicitly tested for [5]. A reliable measurement must therefore address clutter, taper, curved axes, and photogrammetric systematic error together and must do so repeatably enough to serve as a dimensional-control instrument.

Existing point-cloud fitting methods address individual aspects of this problem but do not provide a comprehensive solution. Random sample consensus (RANSAC) fitting [6] is robust to clutter, but its stochastic nature produces different solutions on repeated runs, and its consensus score is ill-posed for tapered cylinders. Deterministic robust estimation techniques overcome the limitations associated with random sampling while maintaining a high degree of resilience to outliers [7,8]. For adequately pre-processed data with few remaining outliers, principal component analysis (PCA) and least-squares estimation perform comparably to, or better than, more elaborate robust estimators [7]. In parallel, generalized-cylinder reconstruction methods developed primarily for laser scanning of forests handle taper and curved axes well [9,10]. Such methods are conceptually well suited to marine masts, since they explicitly handle root flare and branch whorls, which are analogous to the deck-collar flare and the yard attachment bands of a mast. However, they rarely quantify the uncertainty of the recovered centerline and, having been developed for metric laser data, do not address photogrammetric doming.

None of the approaches reviewed in Section 2 is simultaneously deterministic, taper- and clutter-aware, self-referential, uncertainty-quantified, and doming-aware, although all of these properties are required for the reliable geometric assessment of large, partially occluded marine masts.

The contributions of this work are as follows:

1. A multi-instance shared-curvature framework for a candidate bound on systematic photogrammetric distortion. Existing approaches address doming primarily during data acquisition through optimized image geometry or subsequently through model-based surface correction [5]. In contrast, the proposed method exploits redundancy inherent in the reconstructed scene. Because all five masts are reconstructed within the same photogrammetric volume, any reconstruction-induced curvature should appear as a common component, whereas genuine structural deformation is expected to be independent for each mast. By decomposing the estimated bending profiles into shared and mast-specific components, the proposed framework isolates a candidate common-mode component and estimates the contribution of systematic photogrammetric curvature without requiring additional image acquisition, external reference objects, or calibration models. The method is validated using synthetic datasets, where an imposed common component accounting for 49% of the bending-profile variance is recovered as 51%, while the independently imposed mast deflections are preserved to within approximately 10 mm, i.e., within the error floor of the method. Application to the measured dataset provides an approximate bound on possible doming rather than a direct correction (the synthetic test recovers the common mode to within a few percentage points), as the available observations cannot conclusively exclude a common structural loading mechanism; with five masts, the test has low statistical power and is inconclusive on these data. In principle, it is transferable to other scenes containing several similar structures, such as wind farms and groups of industrial chimneys and pipe racks.
2. Identification of spatially correlated reconstruction error as the dominant accuracy-limiting factor and development of a validation protocol that explicitly accounts for it. Accuracy assessments of UAV-derived point clouds commonly rely on synthetic datasets, referred to here as phantoms, generated by adding independent random noise to ideal geometries. The present study demonstrates that this assumption substantially underestimates realistic measurement uncertainty because reconstruction errors are spatially correlated rather than independent. Instead of increasing local point dispersion, correlated errors produce coherent displacement of entire cross-sections, allowing systematic geometric bias to persist while leaving conventional quality indicators, such as per-slice residuals, largely unaffected. Numerical experiments show that phantoms containing only independent noise of the measured magnitude yield a bending-profile root-mean-square error (RMSE) of approximately 7 mm, whereas phantoms whose correlated component is as large as the measured per-slice scatter yield 20–27 mm on average and up to 40 mm at the 90th percentile; with an error of 20 mm root mean square (RMS) on the original phantom, the penalty of correlated over independent error is only about 40% (Section 4.5), so the floor is governed by the amplitude assumed for the correlated component (Section 4.7). Based on these observations, a validation protocol is proposed in which synthetic datasets are iteratively generated until their statistical characteristics—including residual distributions, outlier fractions, angular coverage, and point density—closely reproduce those of the measured point cloud. Remaining discrepancies are then reported explicitly, enabling measurement accuracy to be demonstrated rather than assumed. Application of this protocol revealed that the initial phantom substantially underestimated the noise and contamination levels of the measured data (median per-slice residual 9 mm against 46 mm), leading to a more realistic, empirically supported assessment of the achievable accuracy. The significance of each measured bend is then assessed against the null distribution of the bend that straight phantoms of the same mast produce, which keeps the false-positive rate at the nominal level under the assumed error model (Section 4.11).
3. A deterministic, self-referential, CAD-free pipeline for bending measurement of large, occluded marine masts, cross-validated against an independent sensor. The individual estimation components (PCA axis seeding, iteratively reweighted least squares (IRLS) with Tukey’s biweight, Taubin circle fitting with trimmed refitting, angular-coverage gating, and kernel smoothing) are established, and no algorithmic novelty is claimed for them. The contribution lies in their combination, which provides the five properties set out in Section 2.7 together, whereas each approach reviewed in Section 2 provides at most some of them; the earlier measurement of these masts relied on terrestrial laser scanning and manual registration to a CAD model [2]. A per-height Bland–Altman comparison [11] with an independent TLS survey (RMS differences of 14 and 33 mm and 95% limits of agreement of ±28 and ±65 mm for the transverse and longitudinal components, respectively) provides empirical corroboration of the synthetically derived error floor, except in the longitudinal direction on two masts (Section 4.15).
4. A taper-aware sectioning stage with automatic contamination removal and a quantitative demonstration that point-level robust fitting does not compensate for whole-slice contamination. Throughout this work, *contamination* denotes points that do not lie on the mast surface (rigging, stays, welded attachments, and the deck-collar flare), as distinct from correlated reconstruction error affecting the surface points themselves. The deck-collar flare is rejected by a taper-consistency test and the periodic yard and stay bands by spike detection; both tests use statistic-based thresholds rather than manually selected heights. In an unsmoothed baseline variant of the pipeline, disabling flare rejection increases the estimated bending of one mast from 87 to 410 mm, so that the *straightest* mast would be reported as the most deformed. In the final, kernel-smoothed pipeline, the flare test overlaps with the covariance weighting and the band test: disabling any one or two of them changes the measured bends by at most 9.3 mm, whereas disabling all three raises one bend from 36 to 114 mm (Section 4.12).
5. Explicit delimitation of the results. The quantities that the data do not support are identified: cross-section ovality exceeds the round-ring null level, but it is not reported as a deformation result (the welded-track hypothesis was tested and rejected, and TLS, where it covers most of the circumference, suggests that most of the excess belongs to the reconstructed surface); elastic and permanent deformation cannot be separated from a single static epoch; the largest measured bend (Foremast) could not be confirmed independently because its TLS coverage was insufficient; and the absolute scale could be checked only against the TLS survey, to within approximately 2%, which bounds its effect on the bending estimates (Section 5).

*Scope.* Two methodological choices are deliberate. First, the proposed method is *not* learning-based. Recent networks for parametric primitive fitting [12,13,14,15] outperform classical estimators on segmentation and CAD-reconstruction benchmarks. However, they are trained on synthetic primitive datasets, do not provide a propagated uncertainty (Section 2.4), and are therefore difficult to audit as dimensional-control instruments, which must state the uncertainty of each result. Moreover, after segmentation the point clouds are only lightly contaminated, a regime in which least-squares estimation matches or outperforms more elaborate robust procedures [7]. Second, the method is *not* a stochastic-model-based adjustment. Correlated reconstruction error is bounded *empirically by* a matched phantom and an independent sensor, rather than through a full error-covariance model within the estimator. Incorporating such a stochastic model of the point coordinates into the slice fit is identified as future work.

The remainder of the paper is organized as follows. Section 2 reviews related work on geometric and robust fitting, section-based generalized cylinders, learned primitive fitting, UAV photogrammetry and its systematic errors, and dimensional control in shipbuilding. Section 3 describes the data and formulates the deformation estimand and the pipeline. Section 4 presents the results for the five masts of the *Golden Horizon*, Section 5 discusses their significance and limitations, and Section 6 concludes the paper.

## 2. Related Work

### 2.1. Geometric Fitting of Cylinders and Circles

Estimating a cylinder from 3D points is a nonlinear fitting problem in five parameters—two for the axis direction, two for a point of the axis in the plane orthogonal to it, and one for the radius. Approaches divide into three families [16]. *Accumulator methods* discretize the parameter space and vote: the Hough transform for cylinders [17] and its randomized variants are robust to gross outliers but are limited by memory and by discretization bias and are therefore used mainly for coarse detection rather than metric estimation; related detection schemes cast cylinder extraction as a clustering problem [18]. *Algebraic and minimal solvers* obtain closed-form parameters from small point sets—for example, the direct cylinder solutions of Beder and Förstner from minimal configurations [19] and minimal quadric fits [20]—and are excellent initializers but statistically biased because they minimize an algebraic rather than a geometric residual. *Geometric (orthogonal, or total) least squares* minimizes true point-to-surface distances, which is the maximum-likelihood objective under isotropic Gaussian noise; representative treatments include Nievergelt’s cylinder fitting [21] and the cylindrical regression of Ageev and Sharipov [22]. Because the cylinder problem is separable—for a known axis direction it reduces to a planar circle fit, which admits a closed-form algebraic solution—it is efficiently solved by *variable projection* [23], optimizing over the two axis angles with an inner circle fit at each step. Widely used open-source implementations follow this structure. The present method does not fit a single global cylinder, for the reasons given in Section 2.3.

The inner circle fit is itself a mature topic. The algebraic estimators of Kåsa [24], Pratt [25], and Taubin [26] give non-iterative, closed-form centers and radii. Taubin’s normalization minimizes the statistical bias of the algebraic distance and is the preferred initializer for a subsequent geometric refinement.

### 2.2. Robust and Deterministic Estimation

Real point clouds of engineered structures contain clustered outliers—brackets, flanges, rigging—that a plain least-squares fit cannot tolerate. The dominant robust tool in the vision literature is RANSAC and its efficient variants for primitive detection [6]. RANSAC is effective but has two properties that make it unsuitable for a repeatable dimensional-control instrument. First, it is *stochastic*: its output depends on the random sample sequence and differs between runs unless the seed is fixed, in which case the result is repeatable but depends on an arbitrary seed. Second, its consensus score is ill-posed on a *tapered* surface, where no single radius is correct over an extended segment.

A parallel line of work achieves comparable robustness *deterministically*, through robust statistics rather than random sampling. Nurunnabi et al. combine robust principal component analysis with least-trimmed-squares regression to fit cylinders that survive extreme contamination—on the order of 70% scattered and 25% clustered outliers—and on partially scanned surfaces [7]. Two findings from this literature directly shape the present design. First, deterministic high-breakdown estimators (e.g., the minimum-covariance-determinant estimator and iteratively reweighted least squares with M-estimators [8]) reach RANSAC-level robustness without any randomness. Second, when outliers are *absent*, classical PCA and least squares match or outperform the more elaborate robust estimators [7]. Since the point clouds in the present study are pre-segmented, the pipeline operates in the low-contamination regime in which a PCA seed with a light deterministic robust refinement is statistically appropriate.

### 2.3. Section-Based Generalized Cylinders for Tapered, Curved Structures

A mast is not a cylinder but a *tapered tube with a possibly curved axis—a* generalized cylinder. Fitting one global primitive to such an object confounds taper and bending. The mature pattern for this geometry comes from laser-scanning forestry (terrestrial, mobile, and UAV-borne), where tree stems present the same challenge of a tapering, sometimes leaning or bowed, vertical tube. The established workflow is two-phase [9,10]: a coarse cylinder fit first localizes the stem and gives approximate diameters, after which the cloud is cut into thin horizontal slices whose diameters are re-estimated by circle fitting, and the slice centers are strung into a taper or stem curve. This literature also formalizes several practical constraints that carry directly to masts. The slice-thickness choice is a genuine trade-off: too thick and taper or curvature bias the circle; too thin and the slice degenerates into a ring for which the axis direction is undefined, so slice-level PCA cannot recover it [9]. A slice should be accepted only when its circular edge is sufficiently continuous—i.e., when points cover enough of the angular extent of the ring—which serves as an intrinsic quality gate [10]. Two further contaminations that the forestry pipelines handle explicitly are directly analogous to the marine case. The *root flare* (basal buttress) of a stem produces anomalously large, non-circular cross-sections at the very bottom that are routinely excluded before taper-curve fitting; the mast equivalent is the deck-collar flare where the tube steps through the deck. And *branch whorls* inject clustered off-stem points at quasi-periodic heights that must be suppressed slice-by-slice; the mast equivalent is the set of yard and stay attachment bands—on a square rig such as the *France II*-derived masts studied here, roughly six yards per mast produce attachment bands at regular vertical intervals. Related as-built pipelines in industrial settings connect the same section-fitting idea to piping and plant reconstruction [27], and orientation-specific estimators improve axis recovery from partial coverage [28].

### 2.4. Learned Primitive Fitting

A parallel line of work fits primitives with neural networks: SPFN learns to detect a varying number of primitives under direct supervision [15]; ParSeNet decomposes clouds into parametric surface patches, including B-spline patches [13]; HPNet segments primitives via hybrid semantic and spectral representations [14]; SED-Net learns surfaces and edges jointly and assembles them into a regularized CAD model in one framework [12]. On segmentation and reconstruction benchmarks of synthetic CAD models, these methods outperform classical estimators [12,13,14,15]. Evidence for conditions like those of the present study is, however, limited. The networks are trained and evaluated mainly on synthetic primitive datasets, and their performance degrades on shapes that differ from those seen in training [29]. In the SHREC 2022 benchmark of primitive fitting, the two purely neural classifiers were the methods most affected by uniform and by Gaussian noise, and no evaluation on real scans was available [30]. The methods also return primitive parameters without a propagated uncertainty, whereas the present method needs the covariance of each slice center to weight the centerline (Section 3.7) and to state the statistical uncertainty of the bends. We have not compared a learned method on the present data, for which no representative training set of SfM clouds of tall, rigged masts exists; the classical route is chosen here because it provides the uncertainty and the exact repeatability that the weighting and a dimensional-control report require.

### 2.5. UAV Photogrammetry for Deformation Monitoring and Its Systematic Errors

Unmanned-aerial-vehicle (UAV) imagery processed by structure-from-motion (SfM) photogrammetry has become a standard non-contact tool for deformation monitoring of large infrastructure [3,4] and is attractive for tall, occluded marine rigs because it can reach viewpoints that a deck-based total station or terrestrial scanner cannot. However, SfM introduces a systematic error mode that any deformation claim must confront: *doming* (equivalently *bowling*), a broad-scale, low-order curvature of the entire reconstruction that arises from near-parallel viewing geometry combined with imperfect self-calibration of radial lens distortion [5]. Its amplitude grows with tie-point measurement noise, and it is mitigated by oblique (off-nadir) imagery and well-distributed ground control [5]. The consequence for deformation measurement is direct: a domed reconstruction bends a perfectly straight object into a curve, producing a deformation signal that is indistinguishable from genuine structural bending unless it is explicitly tested for. It was not addressed in the earlier dimensional-control report on these masts, which relied on manual registration to an as-designed CAD model [2].

### 2.6. Dimensional Control in Shipbuilding

Marine dimensional control is, in current practice, CAD-anchored: quality is assessed by registering a scanned point cloud of a fabricated block to its 3D design model and computing deviations at management points [31]. This presupposes a trustworthy as-designed model and a stable registration—precisely the two dependencies that fail for a rigged, in-service mast, where the design model may not reflect the built article and where alignment to it is fragile. The self-referential formulation adopted here removes both.

Registration enters the present work only where two sensors are compared. Learned registration networks align partially overlapping scans without an initial guess; MCI-Net, for example, combines graph-based neighborhood aggregation, progressive exchange of context between feature domains, and dynamic inlier selection and achieves high registration recall on indoor RGB-D (red–green–blue–depth) and outdoor LiDAR (light detection and ranging) benchmarks [32]. Recent advances in intelligent point cloud processing, sensing, and comprehension are summarized in [33]. In the method presented here, the bending estimate does not depend on registration, because it is measured relative to each mast’s own axis and is invariant to a rigid transformation of the cloud; registration is needed only to pair the UAV and TLS profiles at the same height (Section 3.1 and Section 4.15), for which the line through the mast positions suffices. Learned registration would become relevant for aligning the individual TLS stations, which were supplied already registered, or for comparing repeated acquisitions.

### 2.7. Research Gap

None of the approaches reviewed in Section 2.1, Section 2.2, Section 2.3, Section 2.4, Section 2.5 and Section 2.6 provides all of the following for large, occluded, tapered marine masts: (i) *deterministic* and exactly repeatable estimation; (ii) a *taper-aware, curved-axis* model rather than a single cylinder, with explicit removal of the deck-collar flare and the periodic yard and stay attachment bands that otherwise bias the fitted centerline; (iii) a *self-referential* deformation measure that avoids fragile alignment to a CAD model—the very step that limited earlier manual practice; (iv) *propagated uncertainty*, so that a reported bend is accompanied by an uncertainty and can be tested for significance in the absence of an external ground truth; and (v) explicit *separation of structural bending from photogrammetric doming*. Robust-cylinder methods deliver (i) and (iv) for a single primitive; forestry section-fitting delivers (ii) and (iii) but rarely (iv) and does not address (v); CAD-anchored marine surveys address none of them. The intersection of these five requirements defines the contribution of this work.

## 3. Materials and Methods

**Figure 1:**
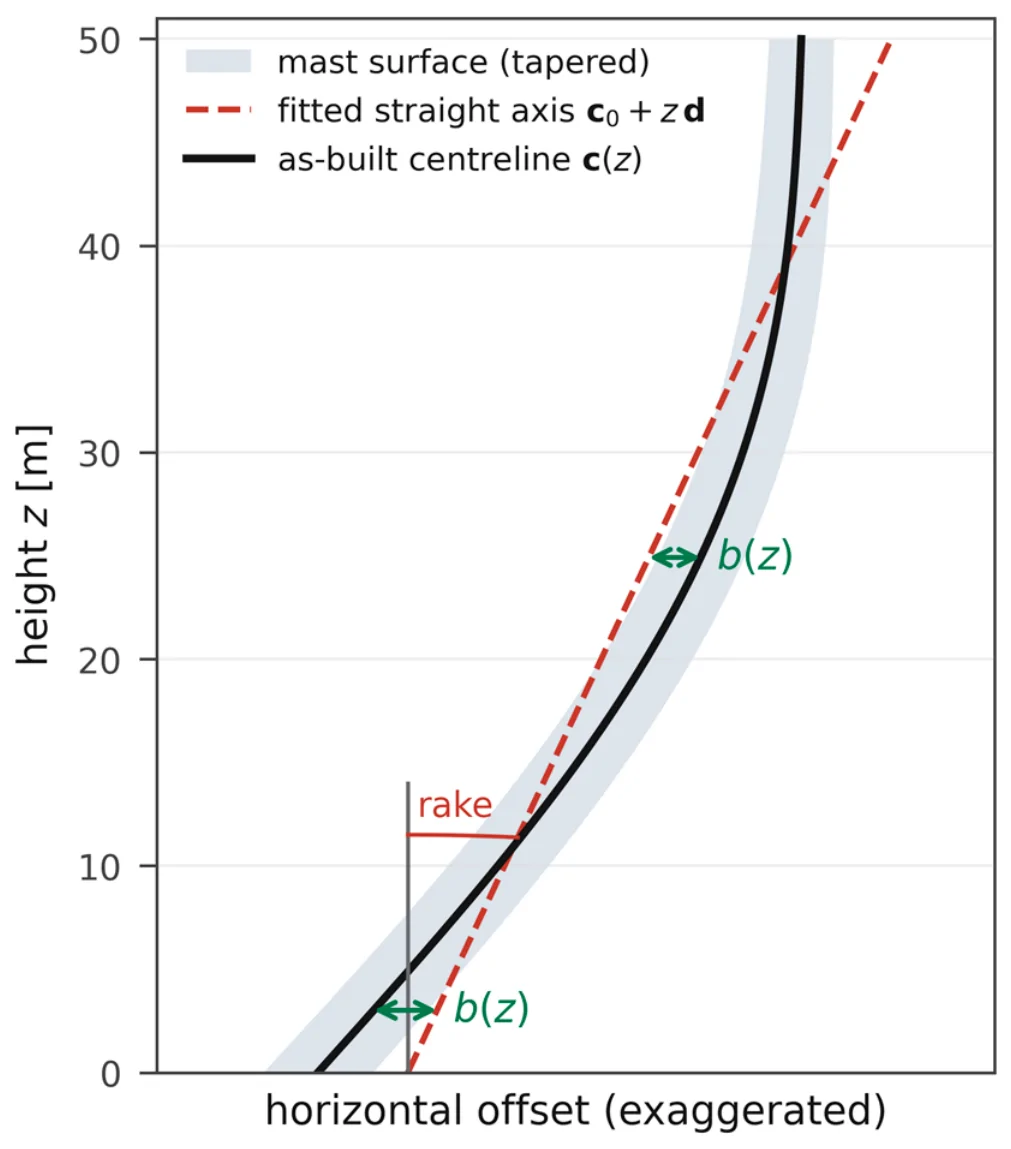
Estimand: bending **b**(*z*) as the departure of the as-built centerline from the straight raked axis fitted to it by least squares (horizontal offsets exaggerated).

**Figure 2:**
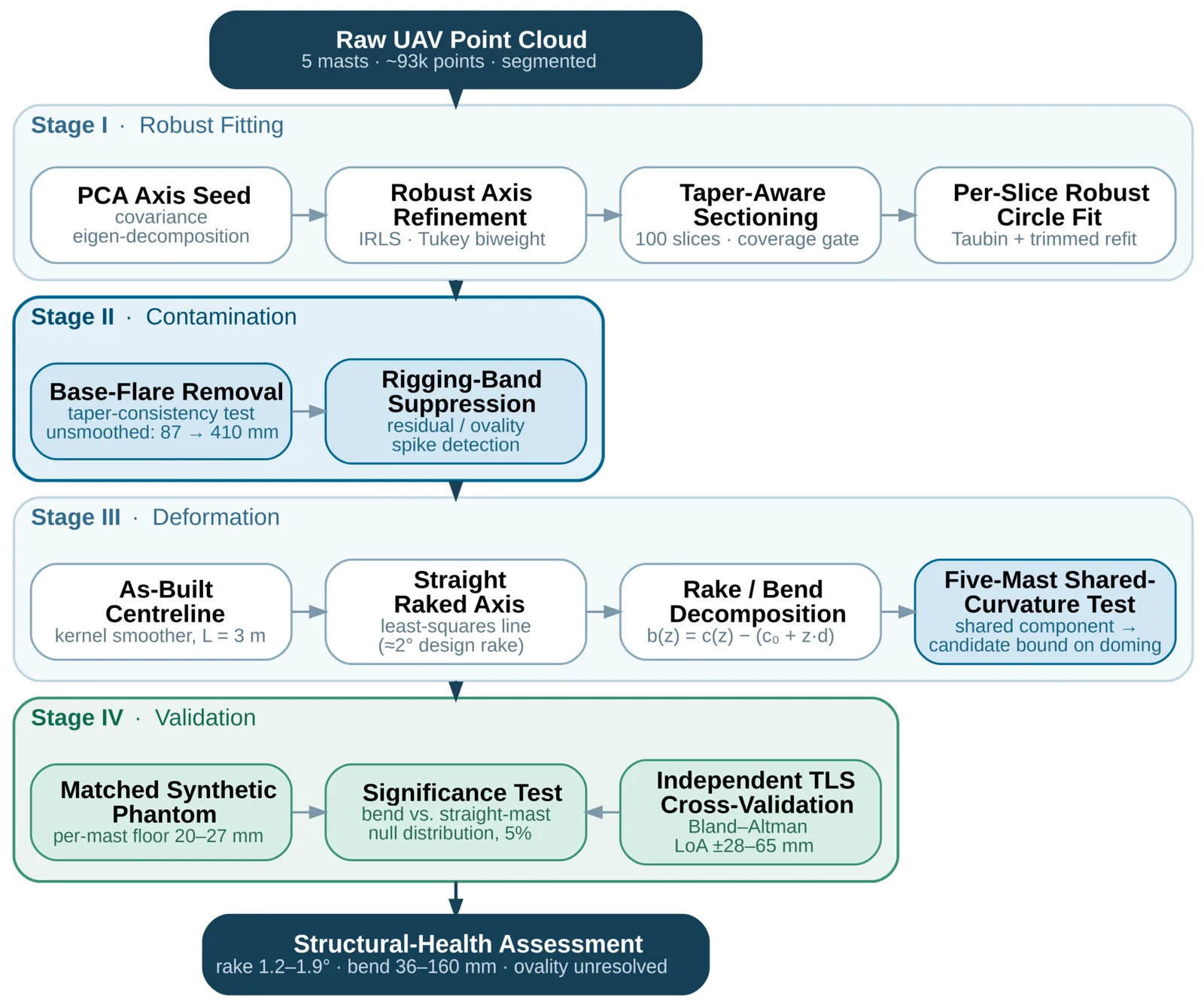
Deterministic processing pipeline, from the raw segmented cloud to a significance-tested structural assessment.

The estimand is illustrated in Figure 1, and the complete pipeline is summarized in Figure 2; its stages are detailed below.

### 3.1. Data Acquisition and Co-Registration

The UAV survey of the *Golden Horizon* was carried out by an external contractor, who supplied the photogrammetric point cloud. According to the survey records available to the authors, the reconstruction was produced in Agisoft Metashape (Agisoft LLC, St. Petersburg, Russia) with ground-control points fixing the metric scale, and the vessel was moored in port during capture. The contractor did not provide the acquisition and processing metadata; the UAV and camera model, the ground sample distance, the number and overlap of the images, the number, distribution, and accuracy of the ground-control points, the checkpoint residuals, and the software version are therefore unknown and are stated as limitations (Section 5). The point cloud itself provides partial indicators of its resolution and noise: the median nearest-neighbor point spacing is 65–68 mm on the five masts, there are 287–486 points per meter of mast height, and the median per-slice radial residual is 46 mm (Section 4.6). The five masts were segmented automatically from the UAV vessel point cloud with the connectivity-based cylinder detector of Araújo and Oliveira [34], which detects cylinders in unorganized point clouds as connected sets of points that project to circles along sampled directions; no manual editing was applied, so the UAV processing chain, from the vessel point cloud to the significance verdicts, is automatic. The released code (Data Availability Statement), implemented in Python 3 (Python Software Foundation, Wilmington, DE, USA) with the NumPy, SciPy, and Matplotlib libraries, starts from the segmented clouds.

The TLS survey used for cross-validation was carried out by the Brodosplit reverse-engineering office in December 2021 with a FARO Focus3D X130 scanner (FARO Technologies Inc., Lake Mary, FL, USA); 16 of 18 scans were registered with reference spheres in the manufacturer’s FARO SCENE software, with the inclinometer switched off [2]. The TLS mast clouds were supplied by the surveyor already segmented. The date of the UAV survey relative to the TLS survey is not documented in these records, so changes to the rig between the two surveys cannot be excluded.

Each point cloud was transformed into a ship frame constructed in the same way from its own geometry. The vertical axis of each frame is the vertical of its cloud: the gravity-referenced vertical of the georeferenced UAV cloud and, for the TLS cloud, the vertical of the registered scan set, because the scanner’s inclinometer was switched off [2]. The longitudinal axis is the line fitted through the horizontal (median) positions of the five masts in the plane perpendicular to that vertical, directed toward the stern-most mast; the transverse axis is perpendicular to both; the origin lies at the centroid of the mast positions; and heights are measured from the deck level of each cloud, taken as the 1st percentile of the heights of its mast points. The two frames are therefore constructed identically but differ by a rotation. They agree in the mast positions (to within 0.1 m transversely and 0.7 m longitudinally; Section 4.8) and in the heading of the line through them, but not in the vertical, and hence neither in the horizontal plane nor, away from the centroid of the masts, in the height datum: the TLS frame is tilted relative to the UAV frame by the angle between the two verticals (3.3°; Section 4.8), which moves individual masts by up to 2.4 m in height (Section 4.15). The term common ship frame is used below in this restricted sense. The bending profiles are insensitive to this tilt, because each is measured relative to its own mast’s straight axis; only the rake, which is referenced to the vertical, is reported from the UAV data alone. Because the TLS clouds contain dense rigging returns (4–7% of the points), they were first cleaned by a band-wise radial filter: in 30 height bands along the robust axis, points whose distance from the axis departs from the band median by more than 2.5 robust scales were removed in three passes. The filter compares each point with the median and the robust spread of the radial distances in its band, so a smoothly oval or deformed cross-section widens the spread and is retained: in simulations of TLS-like masts with an ovality of 0–30%, it removed at most 1% of the true surface points (the Gaussian tails of the 3 mm noise) and 75–100% of the rigging returns and changed the bend by at most 0.3 mm. Only local departures larger than about 2.5 robust scales are removed: a dent 30 mm deep over a 30° sector and 2 m of height lost 57% of its points, and one 60 mm deep all of them, without measurable effect on the centerline (at most 0.1 mm). The cleaned clouds were then processed by the identical pipeline.

No manual reference segmentation of the UAV vessel cloud exists, so the segmentation was checked on the segmented clouds themselves and against the independently segmented TLS clouds. Each UAV mast is continuous along its height, with no gap between consecutive points along the axis larger than 0.09 m, and around it (median slice coverage 100%; Section 3.5). On the four masts with usable TLS coverage, the UAV and TLS segments agree in length to within 1.75 m, so the automatic segmentation does not truncate the masts relative to the independent one. Points more than 0.3 m outside the surface fitted to the retained slices (Section 3.5 and Section 3.6) make up 0.9–3.0% of each mast. Of these, 61–99% lie in the lowest 2 m, where the segment includes the deck immediately around the mast, or in slices that the flare and band tests remove; the rest lie in short clusters beside the mast, which may be fittings or rigging close to it. None lies more than 1.4 m outside the surface, so the segments contain no sails and no yards beyond their attachments. Removing all points more than 0.3 m or more than 0.5 m outside the surface changes the bends by at most 4.0 mm.

### 3.2. Problem Formulation

We model each mast as a generalized cylinder with centerline **c**(*z*) = (*x*(*z*), *y*(*z*), *z*)^⊤^ and radius profile *r*(*z*). The measured points are noisy samples of the surface, contaminated by a minority of outliers. The *estimand* is not the centerline itself but its departure from a straight, raked reference axis (illustrated in Figure 1),

**b**(*z*) = **c**(*z*) − (**c**_0_ + *z* **d**), (1)

where **c**_0_ + *z* **d** is the straight reference axis, fitted to the centerline by least squares (Section 3.7), whose direction **d** gives the as-built rake (designed to be approximately 2^∘^ from vertical for these masts), measured from the vertical of the cloud and therefore including any trim or heel of the moored hull. The linear term therefore absorbs the lean of the mast as built, including any part of its deformation that is linear in height, and the residual **b**(*z*) = (*b_x_*(*z*), *b_y_*(*z*)) is the bending profile, with longitudinal (fore-aft) and transverse (athwartship) components—the physical deformation of interest.

### 3.3. Coarse Axis by Principal Component Analysis

For a slender object, the axis is the dominant direction of the point covariance. With the cloud centered as ${\tilde{p}}_{i}=p_{i}-\overset{‾}{p}$, we form

(2) $$M\;=\;\sum_{i}{\tilde{p}}_{i}\;{\tilde{p}}_{i}^{⊤}\;=\;V\;\Lambda \;V^{⊤},\;\;\Lambda =\mathrm{diag}\left(\lambda_{1}\;\geq \;\lambda_{2}\;\geq \;\lambda_{3}\right),$$

and take the initial axis as **d**^(0)^ = **v**_1_. This estimate is reliable exactly when the axial eigenvalue dominates the two radial ones. For masts approximately 42–54 m high with diameters of approximately 0.7–1.0 m, the aspect ratio exceeds 40:1, so *λ*_1_≫*λ*_2_≈*λ*_3_ and **v**_1_ recovers the whole-mast axis to sub-degree accuracy; the ratio *λ*_1_/*λ*_2_ serves as a built-in confidence in the seed. PCA is used here only for the *whole-mast* axis: on a thin slice the points form a ring, the elongation directions lie in the cross-section, and slice-level PCA cannot return the axis. Slice centers are therefore obtained by circle fitting (Section 3.5), never by slice PCA.

### 3.4. Deterministic Robust Axis Refinement

Starting from **d**^(0)^, the axis is refined deterministically by IRLS. At each iteration, every point receives a weight computed from its radial residual *ρ_i_* = ∥**p***_i_* − proj_axis_(**p***_i_*)∥ − *r*, using Tukey’s biweight,

(3) $$w_{i}\;=\;\left\{\begin{array}{ll} {\left(1-{u_{i}}^{2}\right)}^{2}, & \left|u_{i}\right|<1,\\ 0, & \mathrm{otherwise}, \end{array}\right.\;\;u_{i}=\frac{\rho_{i}}{c\;\hat{\sigma }},\;\;c=4.685,$$

where $\hat{\sigma }$ is a robust scale, estimated as 1.4826 times the median absolute deviation (MAD) of the residuals, *r* is the median point-to-axis distance, and *c* = 4.685 is the tuning constant of the biweight, which gives 95% asymptotic efficiency relative to least squares for Gaussian residuals [35]. Points with |*ρ*_i_| ≥ *c*$\hat{\sigma }$, i.e., |*u*_i_| ≥ 1, receive zero weight. The axis point and direction are then updated as the weighted centroid and the dominant eigenvector of the weighted point covariance, and the procedure is repeated for five iterations. The biweight down-weights residual brackets and rigging continuously and without any random sampling; it is the smooth generalization of a hard Tukey-fence cleaning, of which the binary case *w_i_* ∈ {0,1} is a special instance. Because the input is pre-segmented, the estimate stabilizes within two to four iterations and moves the PCA seed by only 0.05–0.16° on the five masts—it is a safety net, not the primary estimator, consistent with the finding that PCA and least squares are preferred in the low-contamination regime [7]. The refinement is also insensitive to its tuning constant: for *c* between 3 and 9, the measured bends change by at most 2.8 mm and the axis direction by at most 0.21°, the bending-profile RMSE on matched phantoms (Mainmast geometry, six realizations) varies by less than 1 mm (7.0–7.8 mm with white noise and 18.9–19.0 mm with correlated error), and omitting the refinement altogether (PCA seed only) changes the measured bends by at most 3.8 mm.

With the weights held fixed, the axis update of each iteration minimizes the weighted sum of squared point-to-axis distances, Σ_i_ *w*_i_*d*_i_^2^, where *d*_i_ is the distance of point *i* from the axis; each iteration is a weighted total-least-squares line fit. The refinement is therefore an iteratively reweighted line fit in which only the weights come from the cylinder model, through the radial residual *ρ*_i_ = *d*_i_ − *r*. It is not the M-estimator of the cylinder surface, which would minimize Σ_i_ ℓ(*d*_i_ − *r*(*t*_i_)) over the axis and a radius profile *r*(*t*), with ℓ the biweight loss and *t*_i_ the position of point *i* along the axis. The two coincide for a ring sampled uniformly around its circumference, whose weighted centroid is its center, but with partial angular coverage the weighted centroid is drawn toward the covered side. The refined axis is therefore used only to orient the slices, and the centerline is obtained from the per-slice circle fits (Section 3.5), which minimize the radial residual. To quantify the difference, we also fitted the M-estimator of a cone (axis, radius, and linear taper; Tukey biweight with the same *c*, solved by iteratively reweighted Levenberg–Marquardt steps). Its axis differs from the refined axis by 0.04–0.24°, the measured bends change by at most 4.9 mm, and the bending-profile RMSE on 25 matched phantoms (five per mast) is 23.2 mm against 23.1 mm. Because the weights use a single median radius, points toward the ends of a strongly tapered mast receive lower weights, and the median is dominated by the most densely sampled heights. On the five masts, the mean weight of the points in the lowest tenth of the height is 0.61–0.83, against 0.87–0.93 at mid-height. Replacing the single radius with a robust linear radius trend (a Theil–Sen line through the medians of height bins, which does not depend on the point density) changes the lowest-tenth weights only to 0.64–0.82, so the low weights there reflect the base flare and deck fittings rather than the taper; it changes the axis by at most 0.07° and the bends by at most 1.1 mm.

### 3.5. Taper-Aware Sectioning and Circle Fitting with Uncertainty

The cloud is sliced along the *refined axis* (not the global vertical) into bands of thickness Δ. The thickness is chosen such that the taper-induced radius change across a band stays below the point noise *σ*,

|*r*^′^(*z*)| Δ ≲ *σ*, (4)

which is satisfied by a wide margin in the present implementation: each mast is divided into 100 slices of equal thickness, giving Δ ≈ 0.4–0.5 m, for which the taper-induced radius change across a slice is below 3 mm, far smaller than the typical per-slice residual of approximately 46 mm. This thickness is also consistent with forestry practice and with the ring-versus-cylinder trade-off [9,10]. A slice is accepted only if its angular coverage exceeds a threshold (at least seven of twelve 30° sectors must contain points); in the present dataset the median coverage is 100% and the minimum 75%, so no slice is rejected by this gate. For each accepted slice *k* we fit a circle algebraically by Taubin’s method [26], then apply a *deterministic trimmed refit*: points whose radial residual $\left|∥p-{\hat{c}}_{k}∥-{\hat{r}}_{k}\right|$ exceeds 2.5 times the normalized median absolute deviation of the residuals from their median are discarded, and the circle is refit, iterating up to four times. This removes scattered off-ring points (residual noise, sparse spray) and yields center ${\hat{c}}_{k}$ and radius ${\hat{r}}_{k}$ on the dominant ring. It does *not*, by construction, remove a dense one-sided cluster—a welded fitting or a yard parrel occupying one arc of the ring shifts the whole circle rather than producing radial outliers—which motivates the slice-level quality control of Section 3.6. We also propagate a *center covariance* by first-order error propagation,

(5) $$\Sigma_{c_{k}}\;\approx \;{\hat{\sigma }}_{k}^{2}\;{\left(J_{k}^{⊤}J_{k}\right)}^{-1},$$

where **J***_k_* is the Jacobian of the circle residuals at the solution and ${\hat{\sigma }}_{k}^{2}$ the residual variance. A slice observed over a short or lopsided arc yields a large, anisotropic $\Sigma_{c_{k}}$; the covariance thus quantifies, per slice, the reliability of the center estimate.

**Figure 3:**
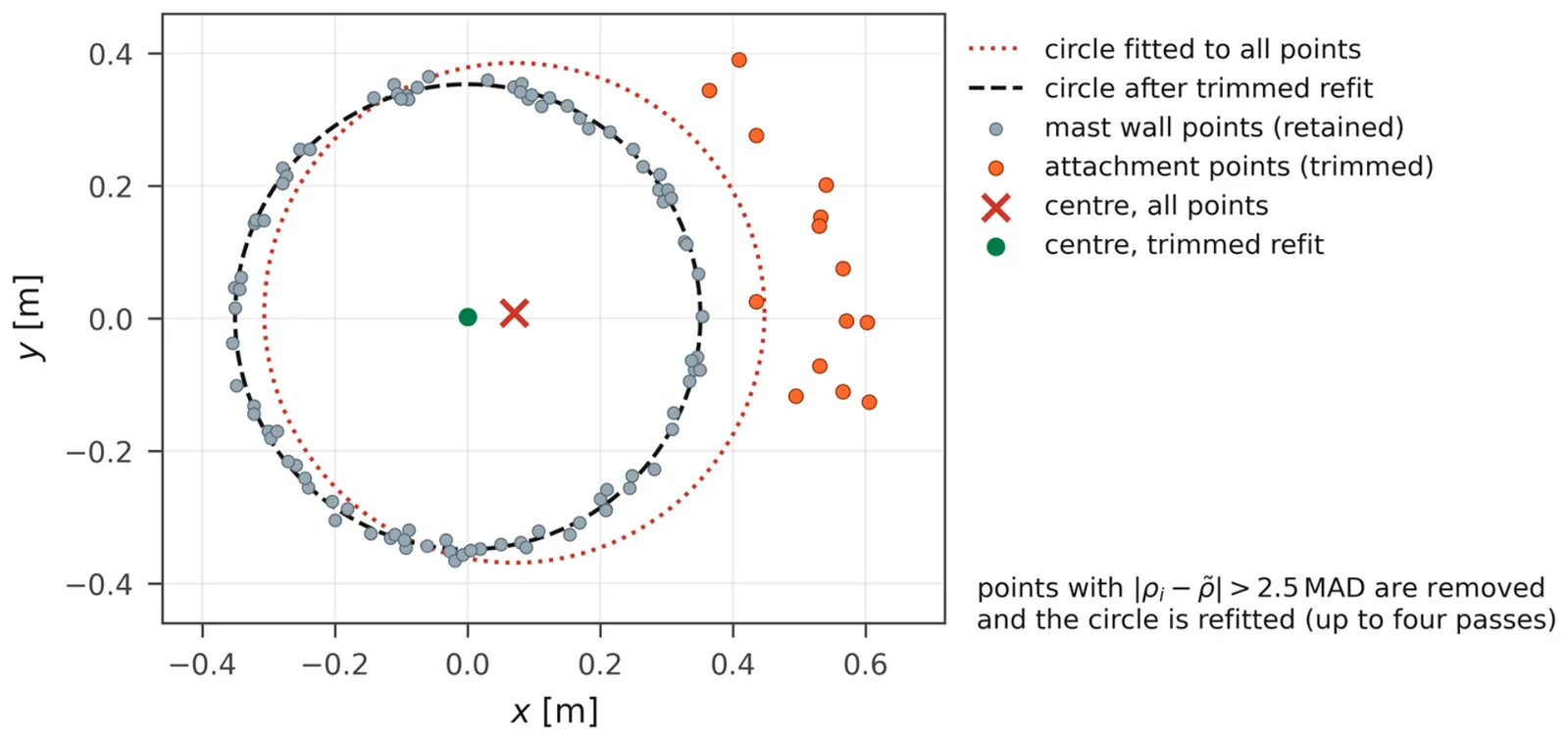
Schematic illustration of the robust trimmed circle fit on a single slice, computed with the pipeline’s circle fit on synthetic points: points lying radially outside the ring are removed, and the circle is refitted.

The effect of the trimmed refit on a single slice is illustrated schematically in Figure 3: attachment points lying radially outside the ring pull the naïve center off-axis, and the trimmed fit removes them and recovers the geometric center. Attachment points lying on the ring itself are not removed in this way and are handled at slice level (Section 3.6).

### 3.6. Slice Quality Control: Base-Flare Removal and Rigging-Band Suppression

Two structural contaminations bias the centerline even after the per-slice trimmed fit, because they act at the level of whole slices rather than individual points, and both are removed by statistic-based tests with fixed constants before the centerline is fitted, rather than by manually selected heights.

*Base-flare (deck-collar) removal.* The lowest meters of each mast flare into the deck fitting and base plate, producing anomalously large and non-circular cross-sections. Left in, these slices anchor the low end of the reference line (Section 3.7) and tilt it, injecting false bending along the entire mast. We fit the radius profile $\hat{r}\left(z\right)$ by least squares on the central portion of the mast (the slices between the 20th and 80th height percentiles, where taper is regular) as a straight taper $\hat{r}\left(z\right)=\alpha +\mathrm{\beta z}$, and reject any contiguous run of bottom (or top) slices whose radius departs from this trend by more than a robust threshold,

(6) $$|\;{\hat{r}}_{k}-\left(\alpha +\beta z_{k}\right)\;|\;>\;\kappa \;\mathrm{MAD}\left(\hat{r}-\left(\alpha +\mathrm{\beta z}\right)\right),\;\;\kappa =3.$$

*Rigging-band suppression.* Yard and stay attachments occur at quasi-periodic heights (on the *France II*-type rig, roughly six yards per mast) and add clustered off-tube points that survive the radial trim. A band manifests as a *local spike*, relative to its vertical neighbors, in the circle-fit residual ${\hat{\sigma }}_{k}$ and in the slice ovality *e_k_* (Section 3.8). We flag as a band any slice whose residual or ovality exceeds a rolling-median baseline (seven-slice window) by more than three robust scales and exclude flagged slices from the centerline fit. Because both tests are threshold-on-statistic rules with fixed constants, the procedure remains deterministic and free of manual height selection; the excluded heights are reported in Section 4.1 and can be compared with the rig layout.

### 3.7. As-Built Centerline and Rake–Bend Decomposition

The centerline is reconstructed as a curve through the *retained* slice centers (Section 3.6), weighted by the inverse of the trace of their center covariance, using a weighted Gaussian kernel smoother of *fixed* bandwidth *L* (here *L* = 3 m). The smoothed center at height *z* is **c**(*z*) = Σ_k_ *K*_k_(*z*)*w*_k_**c**_k_/Σ_k_ *K*_k_(*z*)*w*_k_, with *K*_k_(*z*) = exp[−(*z* − *z*_k_)^2^/(2*L*^2^)] and *w*_k_ = 1/tr **Σ_c_**_k_, where *z*_k_ and **c**_k_ are the height and center of retained slice *k*; *L* is thus the standard deviation of the Gaussian kernel. For densely sampled, evenly weighted slices, the smoother multiplies the amplitude of a sinusoidal bend of wavelength *λ* by *H*(*λ*) = exp(−2π^2^*L*^2^/*λ*^2^). A fixed bandwidth, rather than a cross-validated penalty, is chosen deliberately: it makes the recovered bending profile nearly independent of the slice thickness Δ—which a data-driven penalty does not guarantee, as unsmoothed fine slicing lets the centerline chase per-slice noise and inflates the apparent bend—and it gives an explicit resolved bending wavelength, *λ*_1/2_ = π*L*(2/ln 2)^1/2^ ≈ 5.3*L* (16 m for *L* = 3 m), at which half of the amplitude is retained and below which localized deformation is attenuated and not reported (Section 4.12). Bend estimates vary by at most 21% across Δ∈[0.25,1.25] m at fixed *L*. Weighting lets well-determined slices dominate; smoothing prevents per-slice noise from being mistaken for structural deformation; and the gaps left by removed base and band slices are spanned by the smoother without introducing artifacts. The straight reference axis is then estimated as the least-squares straight line through the smoothed centerline, expressed in the frame of the refined axis, giving **c**_0_ and **d** and, via the estimand definition (Section 3.2), the bending profile **b**(*z*). The linear part reports the rake as an azimuth–elevation pair (and hence which deck quadrant the mast leans into); the residual **b**(*z*) reports longitudinal and transverse bending with the *fitted rake removed analytically* rather than by eye.

### 3.8. Secondary Geometry: Ovality and Inter-Mast Metrics

Cross-sectional *ovality* is estimated per slice from the second angular harmonic of the radial profile (a Fourier-roundness measure, more stable on noisy arcs than a direct ellipse fit): with the fitted center subtracted, *r*(*θ*) ≈ *R*_0_ + *c*_2_cos(2*θ* − *φ*), and

(7) $$e_{k}\;=\;\frac{2\;c_{2,k}}{R_{0,k}+c_{2,k}}\;=\;1-\frac{r_{\min,k}}{r_{\max,k}}.$$

Ovality is meaningful only above the noise and sampling floor: the same estimator applied to a synthetically *round* ring with the slice’s own angular sampling and radial noise gives a null value $e_{k}^{0}$, and only the excess $e_{k}-e_{k}^{0}$ can indicate real flattening or buckling. Where the excess is small or cannot be attributed to an identified cause, ovality is reported as unresolved rather than as deformation. Inter-mast geometry (relative spacing and parallelism) follows directly from the set of five estimated axes, requiring no additional machinery.

### 3.9. Photogrammetric Artifact Control

Two deterministic checks address SfM doming (Section 2.5). First, a shared-curvature test exploits the fact that all five masts were reconstructed together: genuine structural bends are expected to be independent between masts, whereas doming is a property of the reconstruction volume and therefore imprints a common low-order curvature on every mast. The transverse and longitudinal bending profiles are resampled on a common normalized-height grid, the shared component is estimated as their mean profile, and the shared fraction is defined as the fraction of the total bending-profile variance explained by that mean profile. The shared component is reported as a candidate artifact and as an approximate bound on the doming contribution; it is not subtracted from the reported bends, since a common structural cause cannot be excluded (Section 4.4). A positional check correlates the loading of each mast on the shared component with its position along the deck and evaluates the correlation with an exact permutation test over all orderings of the masts. The test requires no additional data, but it is available only when several similar structures are reconstructed together. Second, a scale and straightness check compares the reconstruction with an independent metric reference and, where a provably straight edge exists in the scene, verifies that it is reconstructed straight. In the present data, the inter-mast distances are compared with the TLS survey (Section 4.10); no provably straight edge was available.

### 3.10. Uncertainty Quantification and Validation Without Ground Truth

No ground truth is available for the masts; accuracy is therefore established in four complementary ways. (1) A synthetic phantom, i.e., a simulated mast point cloud with a known bend, rake, taper, noise level, and injected outlier clusters, is passed through the full pipeline; the recovery bias and the RMSE of the recovered bending profile provide the quotable accuracy figures. The resemblance of the phantom to the real cloud is not assumed but verified: its per-slice radial residual, outlier fraction, angular coverage, and point-density profile are compared directly with those of the measured clouds, and the phantom is regenerated until they match (Section 4.6). (2) Repeatability is exact: with no random sampling at any stage, repeated runs are bit-identical, in contrast to the run-to-run variation of RANSAC. (3) The random uncertainty of each slice center is obtained from the covariance of the circle fit; its median over the retained slices, *σ*_stat_, is 5–11 mm for the five masts (approximately 15,000–26,000 points per mast) and is not the limiting uncertainty. The limiting term is the spatially correlated reconstruction error, which is quantified by the RMSE of the recovered bending profile over matched phantom realizations under a correlated error model (specified in Section 4.5); this quantity is referred to as the error floor *ε* (Section 4.5, Section 4.6 and Section 4.7). (4) The bending profiles are compared with those obtained from an independent TLS survey processed by the identical pipeline (Section 4.8). The deformation claim is then falsifiable: a mast is reported as bent only if its total bend *B*, the maximum of the bending magnitude over height, exceeds the total bends that straight masts produce under the same error:

(8) $$B\;=\;\underset{z}{\max}∥b\left(z\right)∥\;>\;B_{0.95},$$

where *B*_0.95_ is the 95th percentile of the null distribution of *B*, obtained by passing straight synthetic masts (zero bend, with the geometry, point density, noise, and correlated error of the respective mast; Section 4.6 and Section 4.7) through the full pipeline. Equivalently, the bend is significant at the 5% level, up to Monte Carlo resolution, if its empirical *p*-value, *p* = (1 + *n*_B_)/(*N* + 1), is below 0.05, where *n*_B_ of the *N* null realizations reach the measured bend; otherwise, it is reported as not resolved. Each mast is tested separately, and the verdicts after a Holm correction for the five masts are also given. The error floor *ε* is not used as the threshold: it is a profile-wide RMSE of one bending component relative to the truth, whereas *B* is the maximum over height of the two-component bending magnitude, which for a straight mast is 1.9–2.5 times as large (Section 4.11). *ε* is retained as the accuracy of the recovered profile. The shared component of Section 3.9 is not subtracted before this test. This criterion, together with the quality control of Section 3.6 and the doming check of Section 3.9, provides the basis for the reported significance verdicts.

### 3.11. Comparison with Terrestrial Laser Scanning

The UAV and TLS bending profiles are compared at the same height in the common frame, over the height range covered by both sensors, after re-referencing each profile to the straight line fitted over that common range, so that both estimate the same quantity. Because each re-referenced profile then has zero mean, the Bland–Altman bias [11] is zero by construction, and agreement is judged from the spread of the differences: the root-mean-square (RMS) difference, the correlation, and the 95% limits of agreement. The Foremast is excluded because its TLS cloud (1930 points) is too sparse to fit. Because both profiles are kernel-smoothed, the per-height differences are strongly autocorrelated; the independent unit is therefore the mast, and confidence intervals for the limits of agreement are obtained by a mast-level block bootstrap.

## 4. Results

Unless stated otherwise, bending figures are per-mast maxima from the kernel-smoothed centerline (bandwidth *L* = 3 m; Section 3.7), reported in millimeters after the fitted straight axis (the rake) is removed.

### 4.1. Dataset and Pipeline Execution

**Figure 4:**
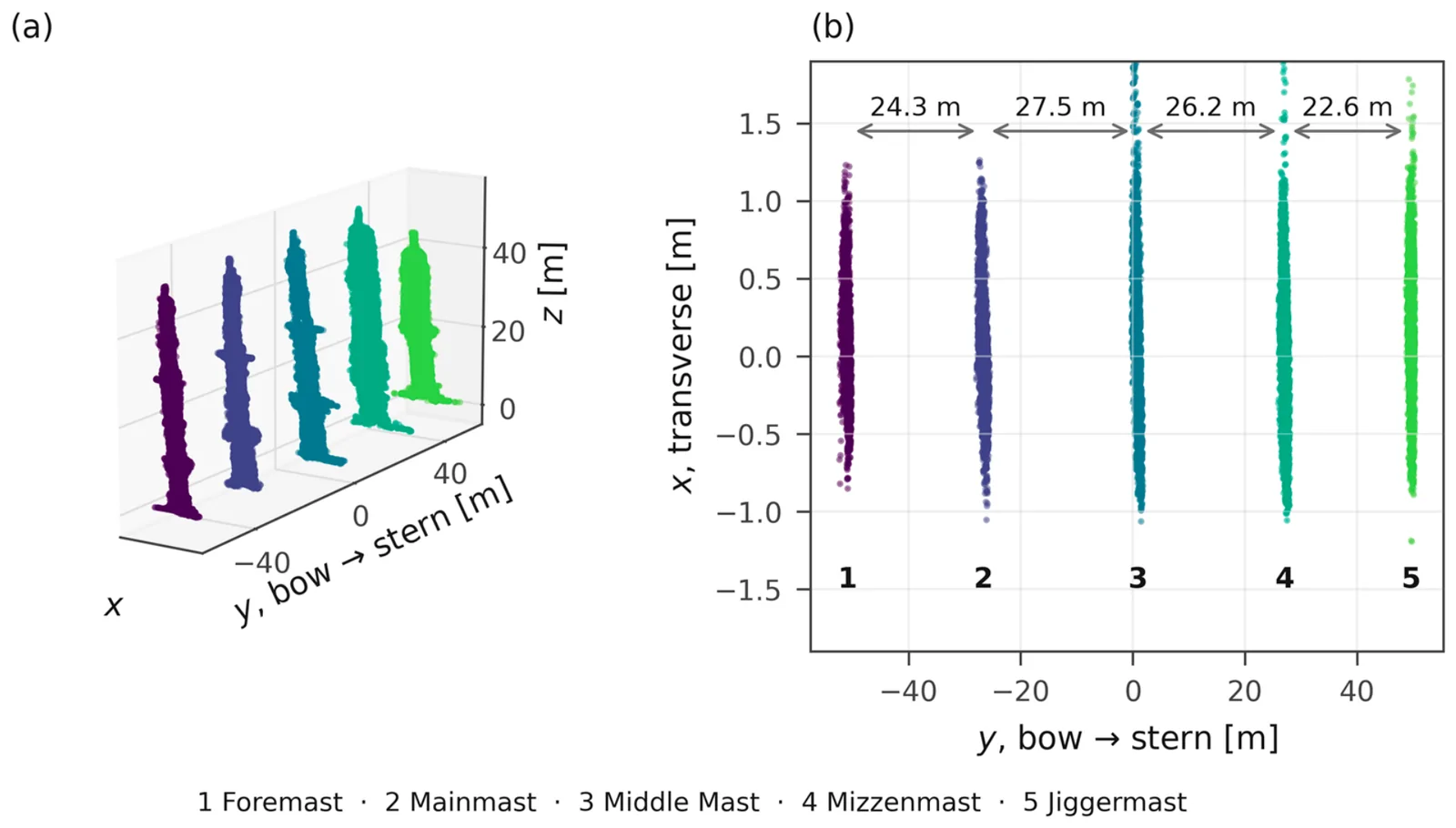
Segmented unmanned aerial vehicle (UAV) point cloud, common ship frame: (**a**) three-dimensional (3D) view; (**b**) plan view with inter-mast spacing (transverse axis exaggerated).

**Figure 5:**
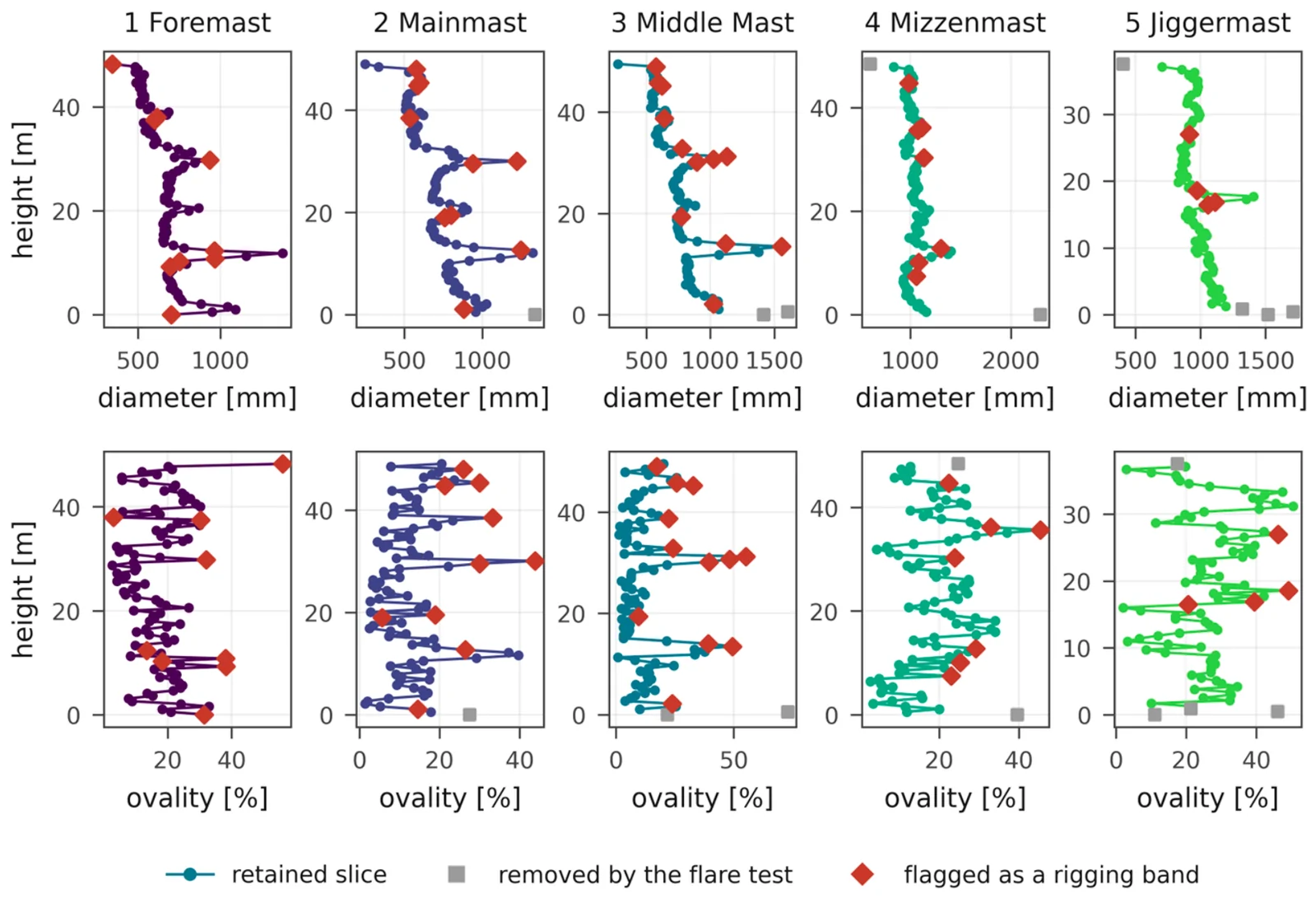
Slice quality control on the UAV clouds (100 slices per mast), bow to stern: fitted diameter (**top**) and ovality (**bottom**) of each slice. Line colors identify the masts; gray squares mark slices removed by the flare test, and red diamonds mark slices flagged as rigging bands; heights are measured from the lowest accepted slice.

The five segmented mast point clouds, co-registered into the common ship frame, are shown in Figure 4. The method was applied to all five (14,752–25,834 points each; 93,393 points total). Every stage is deterministic: for identical input, the pipeline returns bit-identical parameters on repeated execution. The principal-component seed was well conditioned on all masts (axial-to-radial eigenvalue ratio 728–2210), confirming the high-aspect-ratio regime in which the dominant eigenvector recovers the axis to sub-degree accuracy. After sectioning, the slice quality control removed 0–3 contiguous base slices (deck-collar flare) and at most one top slice per mast and flagged 4–12 rigging-band slices per mast (Figure 5). Excluding the end slices, the flagged slices form three to eight bands per mast at irregular vertical intervals (median ≈ 7 m), which is of the same order as the yard spacing expected on the four square-rigged masts. As the yard heights were not available for a direct comparison, this agreement is indicative rather than confirmatory.

### 4.2. Impact of Base-Flare Removal and of the Smoothing Scale

**Figure 6:**
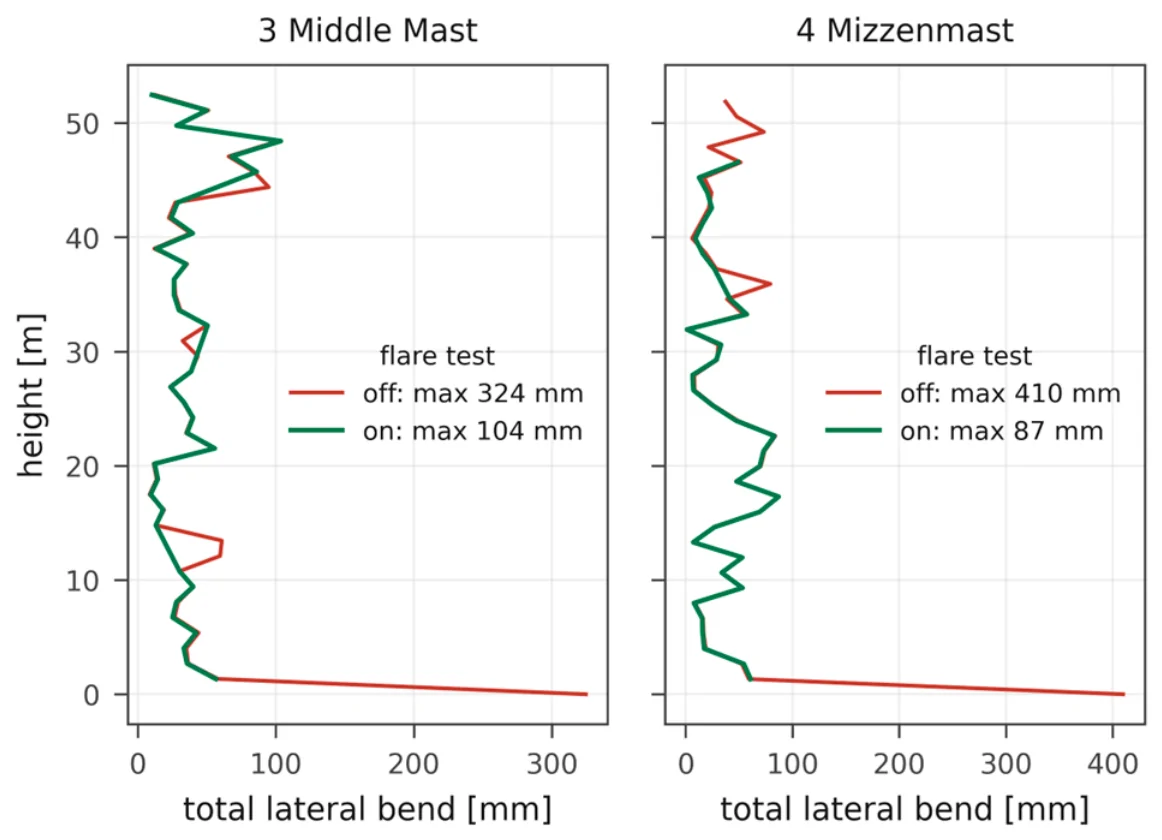
Effect of base-flare removal in the unsmoothed baseline pipeline (Middle Mast and Mizzenmast): total lateral bend with the flare test disabled and enabled, plotted against height above the lowest accepted slice.

When the centerline is not smoothed, base-flare removal has the largest single effect on the estimated bends. To expose the mechanism, the flare test was disabled in an unsmoothed baseline variant of the pipeline, in which the straight reference line is fitted directly to the centers of 40 slices per mast without kernel smoothing. For the two masts with a pronounced base flare, the apparent bend then increases from 87 to 410 mm (Mizzenmast) and from 104 to 324 mm (Middle Mast), because the flared slices anchor and tilt the straight reference line (Figure 6). For the masts with clean bases, the change is much smaller (Foremast 218 to 257 mm, Mainmast 132 to 135 mm, Jiggermast 115 to 131 mm). The synthetic ablation (Section 4.5) reproduces this behavior on a phantom with known geometry. Without flare removal, the straightest mast (Mizzenmast) would therefore be reported as the most deformed. The kernel-smoothed operating pipeline is far less sensitive to the flare (changes of at most 3 mm when the flare and band tests are disabled), because the covariance weighting also down-weights the flared slices; only when the flare test, the band test, and the weighting are all disabled does the Mizzenmast bend rise from 36 to 114 mm (Section 4.12). This redundancy is a property of the final pipeline rather than evidence that the flare is harmless.

The choice of smoothing scale influences the stability of the estimated centerline curvature. Without smoothing, fine slicing lets the centerline chase per-slice noise, and the maximum bend is unstable (e.g., Foremast rises 218→591 mm as Δ falls 1.25→0.25 m). The fixed-bandwidth kernel smoother (*L* = 3 m) largely removes this dependence—with 40, 100, and 200 slices per mast (Δ ≈ 1.25, 0.5, and 0.25 m), the bends change by at most 21% (Foremast 137/160/160 mm; Middle Mast 36/45/54 mm; Mizzenmast 39/36/38 mm)—at the cost of an explicit, reported resolution scale below which localized bending is not resolved. All reported bends are the smoothed values at Δ ≈ 0.5 m (Table 1). The dependence on *L* itself is smooth and monotone: for *L* = 2, 3, 4, and 5 m, the Foremast bend is 176, 160, 147, and 136 mm, the Mainmast bend is 99, 89, 77, and 67 mm, and the Mizzenmast bend is 48, 36, 30, and 23 mm, i.e., the bend decreases with increasing bandwidth without qualitative change. *L* = 3 m was chosen a priori to bridge the gaps left by removed slices and to suppress localized features such as residual rig-band effects (Section 4.13). The monotone decrease with *L* does not, however, distinguish noise suppression from attenuation of genuine bending, and Section 4.12 shows that *L* = 3 m does not retain bending at the inter-yard scale (≈7–8 m), which it attenuates to at most 7% of its amplitude; it retains half of the amplitude at a wavelength of 16 m and 82% at 30 m. The reported bends therefore describe deformation at wavelengths of roughly 16 m and longer, which carry most of the bending power of the measured profiles. The error floor (Section 4.7) and the null distribution (Section 4.11) are derived with the same bandwidth, and Section 4.13 repeats both for *L* = 2–5 m.

### 4.3. Rake and Bending Profiles

**Figure 7:**
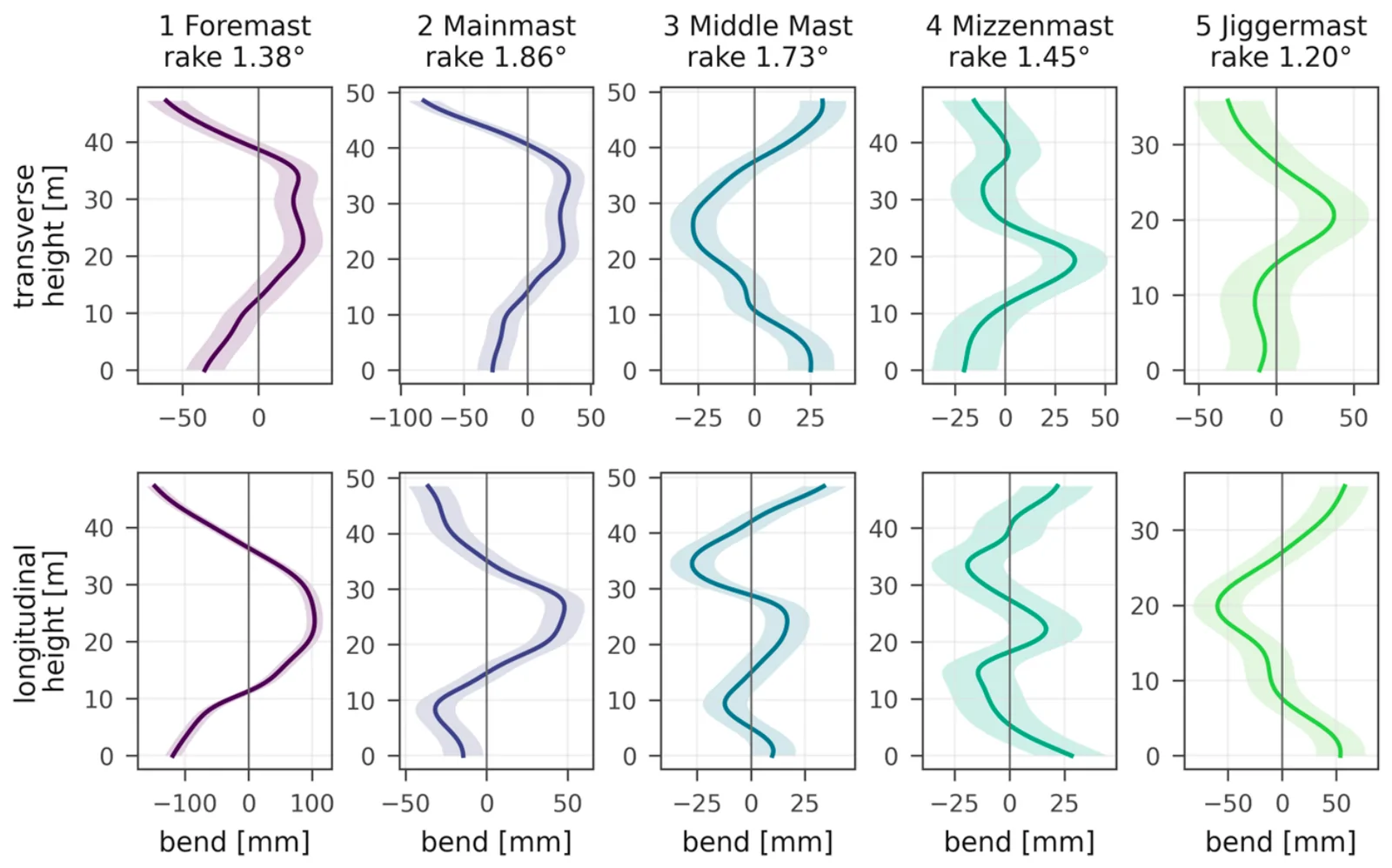
Kernel-smoothed bending profiles (*L* = 3 m, rake removed), bow to stern: transverse (top) and longitudinal (bottom) components, each mast in the same color as in Figure 5. The shaded band, in the color of the mast, is ±2*σ*_stat_ (median slice-center uncertainty) and does not include the correlated-error floor.

**Figure 8:**
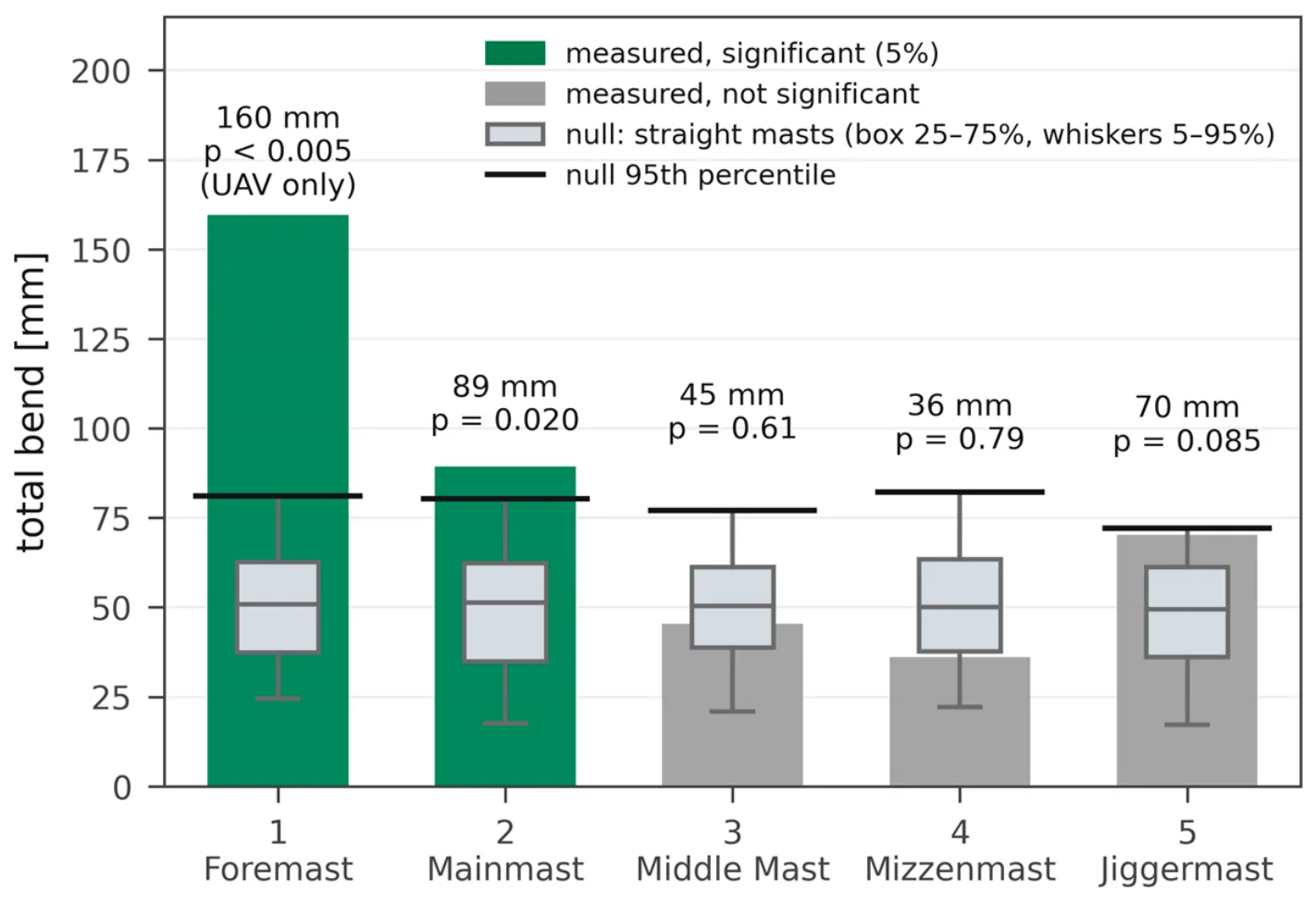
Maximum bend of each mast (bar; green, significant at the 5% level) against the null distribution of the total bend of 200 straight synthetic masts matched to that mast (box: quartiles; whiskers: 5th–95th percentiles) and its 95th percentile (horizontal line), with the empirical *p*-value of the measured bend. The Foremast bend (marked “UAV only”) could not be confirmed by terrestrial laser scanning (TLS; Section 4.8).

The fitted axis rake is 1.2–1.9° from vertical on all five masts, consistent with the intended aft design rake of approximately 2° (the comparison with TLS in Section 4.8 suggests an uncertainty of up to about 0.8° in the absolute rake) and stable under trimming and smoothing. The full profiles (Figure 7) are decomposed into transverse (athwartship) and longitudinal (fore-aft) components; the Foremast bend is dominated by its longitudinal component (148 mm, against 61 mm transverse). The random slice-center uncertainty *σ*_stat_ is small (5–11 mm), but it is not the limiting uncertainty; the realistic per-mast error floor is 20–27 mm on average and 29–40 mm at the 90th percentile (Section 4.7). Applying the decision rule of Section 3.10, a bend is significant if it exceeds the 95th percentile of the total bends that straight masts of the same geometry produce under the same error (Section 4.11). On this basis, the Foremast (160 mm, *p* < 0.005) and the Mainmast (89 mm, *p* = 0.020) show significant bending at the 5% level, whereas the bends of the Jiggermast (70 mm, *p* = 0.085), the Middle Mast (45 mm, *p* = 0.61), and the Mizzenmast (36 mm, *p* = 0.79) are not resolved (Figure 8). The two significant results are supported differently. No straight phantom reached the Foremast bend, but it rests on the UAV data alone, because its TLS coverage is too sparse to fit (Section 4.8), and 148 mm of it is longitudinal, the component that also dominates the candidate shared mode (Section 4.4). Over the common height range, where UAV and TLS give maxima of 76 and 70 mm, the Mainmast bend agrees with TLS in its transverse component (RMS difference 11 mm, *r* = 0.84), which carries most of it, but less closely in its longitudinal component (35 mm, *r* = 0.63; with the tilt-corrected height pairing of Section 4.15, 15 mm transversely and 28 mm longitudinally), so TLS corroborates it only in part. It exceeds the null threshold of its mast (80 mm) by only 9 mm and is not significant after a Holm correction for the five masts. The Jiggermast falls just short of its threshold (72 mm), and its verdict depends on the error model: it is significant under seven of the fourteen models of Section 4.7. With a Bonferroni correction over all slice-wise comparisons, which raises the threshold for random error to ≈3.8*σ*_stat_ (20–43 mm for the five masts; 413 retained slices, two-sided α = 0.05), the dominant per-mast bends also remain significant against random error; the correlated error, however, remains the limiting term, and it is the one that the null distribution of Section 4.11 represents.

### 4.4. Five-Mast Shared-Curvature Test for Candidate Doming

**Figure 9:**
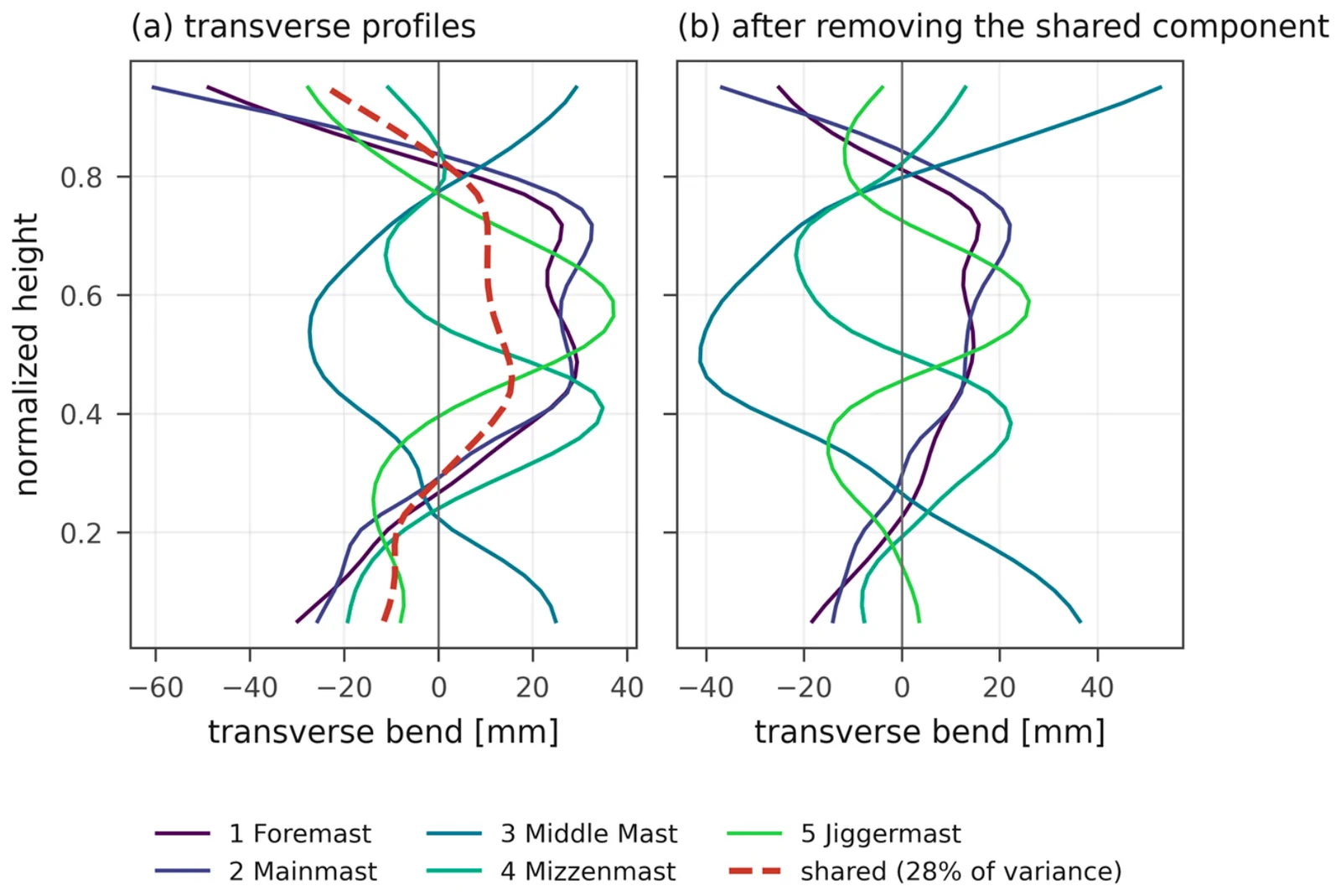
Five-mast shared-curvature test for the transverse component: (**a**) bending profiles on a normalized height axis with the shared (mean) component; (**b**) the mast-specific residuals after its removal.

A curvature common to several independently fitted masts is a candidate reconstruction-wide artifact. After trimming, the shared component accounts for 28% of the transverse bending-profile variance and for 14% of the longitudinal variance (Figure 9). The longitudinal (fore-aft) bending is therefore largely mast-specific, whereas a larger share of the transverse bending is common to all masts. A mast-specific component is less exposed to a reconstruction-wide artifact, but it is not thereby free of error (Section 4.8). We tested whether the loading of each mast on the shared component varies with its position along the deck, as a reconstruction-wide distortion would tend to, using an exact permutation test over the 120 orderings of the five masts. For the transverse component, which carries the larger shared fraction, the loading shows no trend (*r* = −0.29, *p* = 0.65). For the longitudinal component, the loading decreases monotonically from bow to stern (*r* = −0.96, *p* = 0.017); this trend is, however, carried by the Foremast, whose large longitudinal bend dominates the mean profile, and it disappears when the shared component is recomputed without the Foremast (*r* = +0.56, *p* = 0.50). The Foremast also dominates the shared profile itself: its contribution alone (27 mm at its peak) is as large as the peak of the longitudinal shared profile (27 mm), which falls to 16 mm when the Foremast is excluded, and excluding it lowers the shared fraction from 28% to 16% (transverse) and from 14% to 13% (longitudinal). With five masts, the test has little statistical power (the smallest attainable exact *p*-value is 2/120 ≈ 0.017), and the result is inconclusive: it neither confirms doming nor excludes a common structural or environmental cause (identical rig, common berth, and thermal state). The shared component is therefore treated as a *candidate* common-mode artifact and reported as a bound; it is not subtracted from the bends in Table 1, which thus represent the upper bound, whereas subtracting it would give a lower bound on the mast-specific bending.

### 4.5. Synthetic Validation: Accuracy and Failure Modes

**Figure 10:**
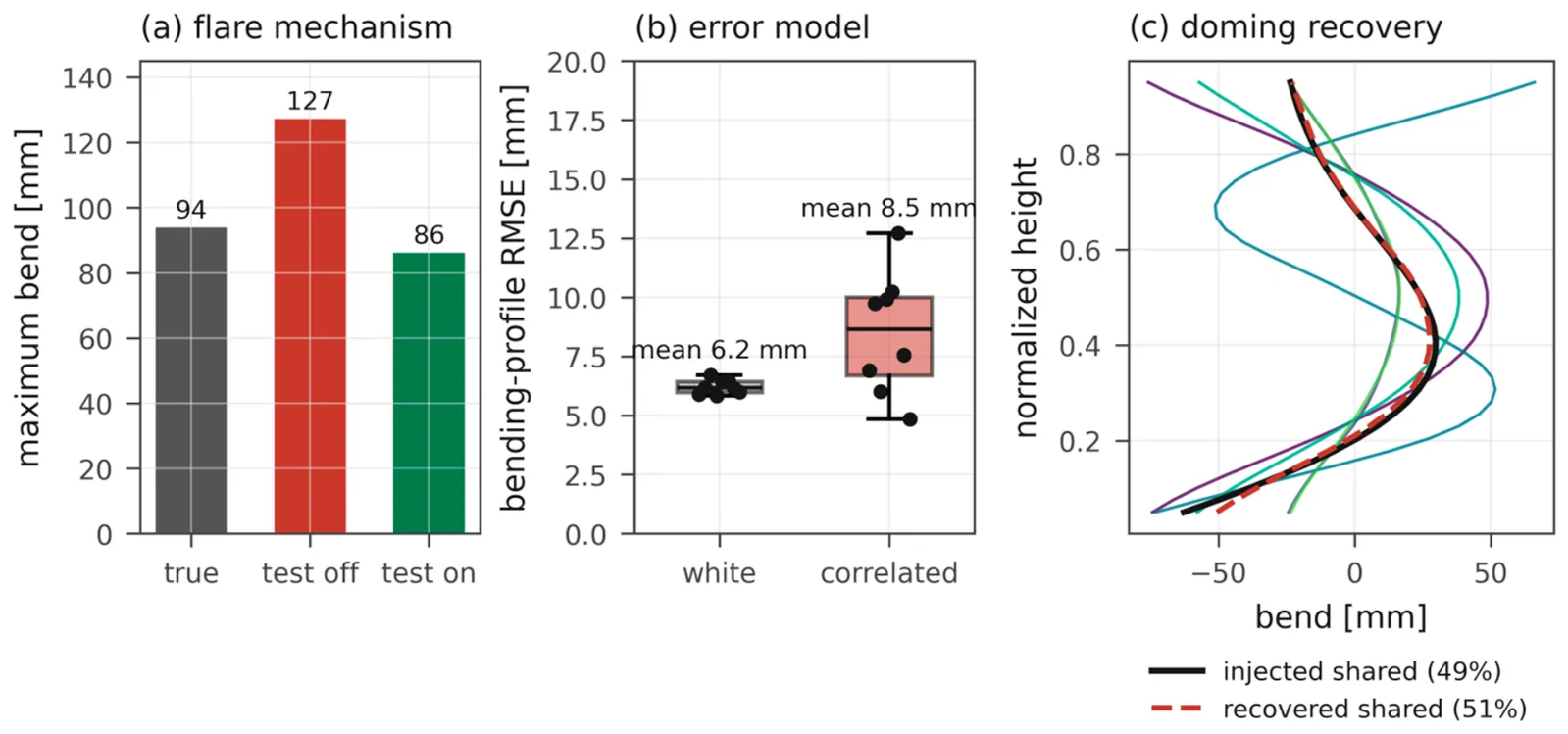
Extended synthetic validation: (**a**) flare mechanism on a phantom with a true bend of 94 mm (unsmoothed baseline pipeline): true bend (gray) and bends recovered with the flare test off (red) and on (green); (**b**) bending-profile root-mean-square error (RMSE) under white (gray) and spatially correlated (red) error of the same magnitude (eight seeds); (**c**) recovery of an injected shared curvature: recovered per-mast profiles (thin lines, mast colors as in Figure 5) and the injected (black) and recovered (red dashed) shared components.

Accuracy was quantified on synthetic phantoms with a known curved axis, matched noise, one-sided clutter, and a base flare. Three findings emerge (Figure 10). (i) *Flare mechanism*: on the phantom, disabling flare rejection inflates the recovered bend from a true 94 mm to 127 mm, and enabling it returns 86 mm, reproducing the behavior observed on the real Mizzenmast and Middle Mast. This is shown with the unsmoothed baseline pipeline, the same variant used for the real-data demonstration in Figure 6. (ii) Error model: under white surface noise of 20 mm, the bending-profile RMSE is ≈6 mm, and under smooth spatially correlated error of the same magnitude (the realistic model for SfM), it is ≈8.5 mm on average, with a wider seed-to-seed spread (5–13 mm) because the outcome depends on the realized error field. The correlated component dominates as its amplitude grows: a phantom matched to the measured cloud statistics, whose correlated field is 45 mm RMS, gives ≈21 mm (Section 4.6), and the per-mast floor has means of 20.0–27.1 mm (Section 4.7), whereas the same phantoms with independent noise of 45 mm instead give ≈7 mm. Correlated error is retained as an apparent shape, whereas white noise averages out in the slice center. (iii) *Doming recovery*: on a synthetic five-mast scene with a known injected common curvature (49% shared fraction) plus independent per-mast bends, the shared-curvature test recovers a 51% shared fraction, validating the test while showing that it slightly overestimates the common mode; the independent per-mast bends are preserved to ≈10 mm, i.e., within the error floor of the method. Taken together with the TLS cross-validation (Section 4.8), the synthetic evidence gives a realistic error floor of approximately 20–40 mm, depending on the mast and on whether its mean or its 90th percentile is used (Section 4.7), rather than the ≈6–7 mm obtained with white noise.

*Correlated error model.* The correlated field displaces the points horizontally, with no vertical component. It is the sum of five modes sin(*k*_z_π*z*/*H* + *k*_θ_*θ* + *φ*), with (*k*_z_, *k*_θ_) ∈ {(1,0), (2,0), (3,0), (1,1), (2,1)}, where *z* is the height, *H* the mast height and *θ* the angle around the ring; the amplitudes are Gaussian and the phases *φ* are uniform, drawn independently for the two horizontal components, and the field is rescaled to a prescribed RMS per component. The three *k*_θ_ = 0 modes translate whole slices, with vertical wavelengths of 2H, H, and 2*H*/3 (28–107 m for these masts); their autocorrelation falls to 1/e at a lag of 7.7–9.8 m, which we take as the correlation length of the field. The two *k*_θ_ = 1 modes, which carry 40% of the variance, deform the rings without moving their centers. In expectation, the field is isotropic in the horizontal plane. Its RMS is 20 mm per component in the error-model experiment above and 45 mm in the matched phantoms of Section 4.6 and Section 4.7, chosen equal to the measured median per-slice residual (45.6 mm). This is a deliberately pessimistic choice: the matched phantoms also carry incoherent noise of 40 mm RMS and scattered off-surface points, which by themselves reproduce the measured per-slice statistics (Section 4.6), so the correlated component is assumed to be as large as the whole observed per-slice scatter in addition to it. These choices are not identified by the data; Section 4.7 quantifies how the floor depends on them.

### 4.6. Representativeness of the Phantom and Transfer of Accuracy

**Figure 11:**
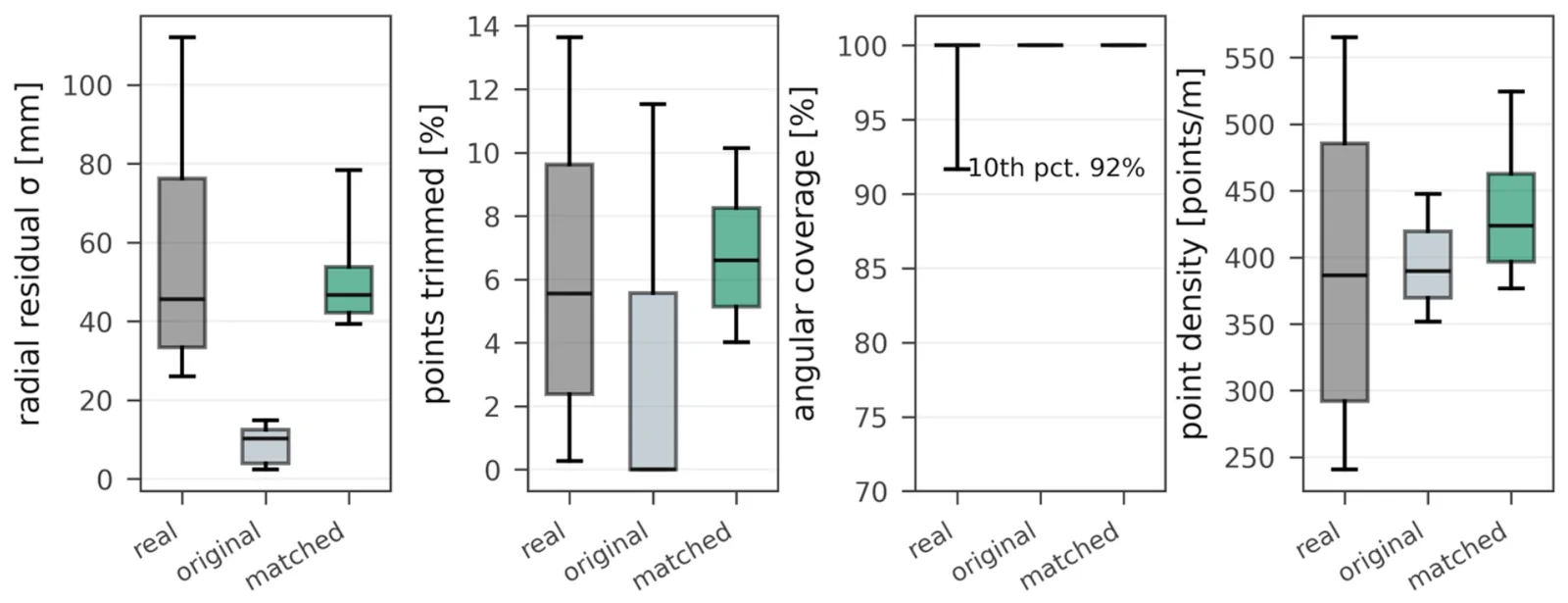
Per-slice statistics of the real UAV clouds, the phantom first used (original), and the phantom regenerated to the measured statistics (matched); see Table 2. Boxes show quartiles and whiskers for the 10th–90th percentiles.

An accuracy figure derived on a phantom transfers to the real masts only if the phantom resembles them in the statistics that govern fitting accuracy. We therefore compare the two directly (Figure 11, Table 2). The phantom initially used was substantially milder than the data: its per-slice radial residual was 9 mm against the measured 46 mm, it carried no heavy tail (90th percentile 11 mm against 112 mm), and only 0.2% of its points were trimmed as outliers against 5.6% in the real clouds. The point density matched, but the phantom was not representative in the statistics that govern fitting accuracy.

Regenerating the phantom to match the measured statistics (surface noise and contamination raised, and a scattered heavy-tailed off-surface component added, since the real outliers are not confined to attachment bands) more than doubles the transferred error: the correlated-error RMSE rises from about 9 mm to about 21 mm (range 11–32 mm over seeds). The increase comes from the larger correlated field (45 against 20 mm RMS) rather than from the added incoherent scatter: each slice center averages over hundreds of points, so incoherent per-point scatter largely averages away, and the same matched phantom with independent noise only gives about 7 mm (Section 4.5). The limiting term remains the *spatially correlated* component, which appears in the per-slice residual only through its ring-deforming part: its slice-translating part, the larger share, displaces a slice rather than scattering it. Matching the residual spread is therefore necessary but not sufficient: the amplitude of the correlated component, which the per-slice statistics do not identify, sets the floor (Section 4.7).

Three discrepancies remain after matching. The real clouds retain a heavier residual tail (90th percentile 112 mm against 75 mm), and their rings show mild angular-coverage gaps behind rigging (10th percentile 92% against 100% for the phantom). Within the range tested, recovery accuracy was insensitive to both; applying this finding to the real data is, however, an extrapolation. Third, under correlated error, the flare test removes the lowest 3–5 m of a phantom, because the correlated field modulates the fitted radius, whereas it removes at most three slices (about 1.5 m) of the real masts, so the phantom profiles and the floors derived from them span a slightly shorter height range than the real ones. The correlated-error floor derived from the matched phantom (≈21 mm on average; per mast in Section 4.7) is therefore adopted, supported by a direct comparison of phantom and measured statistics.

### 4.7. Per-Mast Error Floor

A single error floor implicitly assumes that the five masts pose the same estimation problem. They do not: they differ by 12 m in height and by a factor of six in taper (the Mizzenmast is nearly a straight tube; the Mainmast and Middle Mast taper most strongly), and both quantities affect the slice count, the angular coverage, and the radius-trend fit that drives flare rejection. We therefore repeated the phantom study *per mast*, giving each phantom the height, base radius, taper, and point density of the mast it stands for, in addition to the measured per-slice noise statistics.

**Figure 12:**
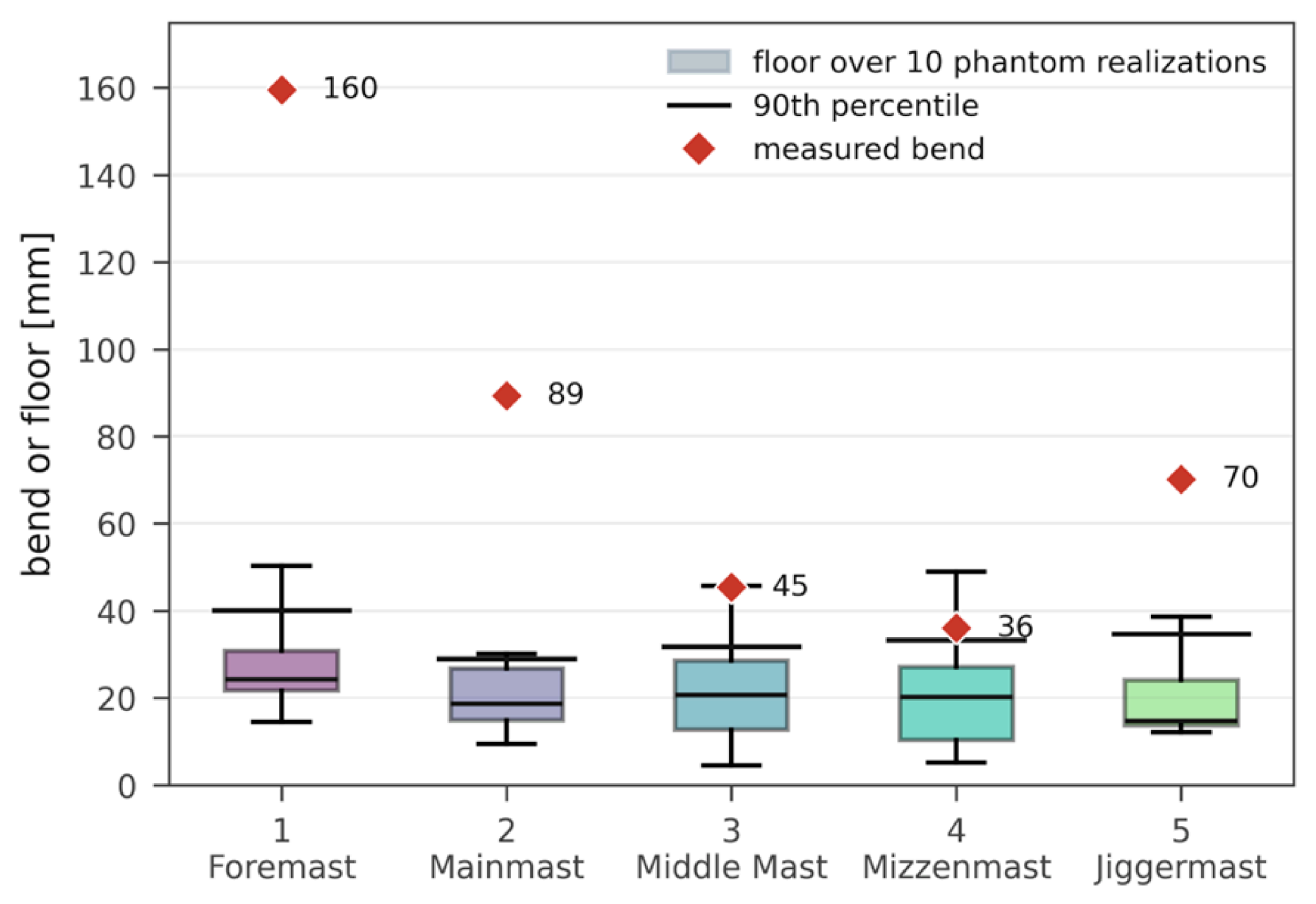
Per-mast error floor over 10 phantom realizations (box: quartiles; whiskers: range) and its 90th percentile (solid line), with the measured bend (diamond) for scale; the floor is the accuracy of the recovered profile, not a significance threshold (Section 4.11).

The floors are similar (mean 20.0–27.1 mm; Table 3 and Figure 12) and close to the ≈21 mm of the generic matched phantom (Section 4.6). However, each floor is a *distribution* rather than a single value (90th percentiles 29.0–40.1 mm), and neither statistic is a significance threshold: the verdicts follow from the null distribution of the total bend (Section 4.11).

The floors measure the accuracy of the recovered profile, not the size of the bend that a straight mast can appear to have: the total bend of the same phantoms without a bend has means of 48–51 mm and 95th percentiles of 72–82 mm (Table 3, Section 4.11), well above the floors. The floor estimates themselves carry sampling uncertainty: repeating the study with 30 phantom realizations per mast (Table 3 uses 10) changes the floors by at most 7 mm, with bootstrap 95% confidence intervals of approximately ±3–4 mm on the means and ±4–11 mm on the 90th percentiles.

*Sensitivity to the error model.* The floors above depend on the correlated-error model of Section 4.5, whose spatial covariance cannot be derived from the data: a phantom can be matched to the per-slice statistics of the real cloud, but not to the full covariance of its SfM error. We therefore recomputed the per-mast floors (10 realizations per mast, 50 per model) with the default field replaced by stationary Gaussian-process fields along the height, with squared-exponential correlation lengths *l* of 1–40 m, and varied the amplitude, the anisotropy, and the share of the angular component (Table 4). The floor depends most on the amplitude: its mean rises from 10 to 29 mm as the RMS of the field increases from 15 to 60 mm per component. It depends less on the correlation length, with means of 16–23 mm for *l* = 1–40 m and a maximum near *l* = 5–10 m; we attribute this to the 3 m kernel, which averages shorter errors, and to the straight reference line, which partly absorbs longer ones. Concentrating the error in the plane of the bend raises the mean floor by 19% relative to the isotropic field with *l* = 10 m (anisotropy 2:1), and a field that only translates slices raises it by 30%. The default field, with a mean floor of 22 mm, lies in the upper half of these models; the 60 mm amplitude, the 2:1 anisotropy, the translation-only field, and the fields with *l* = 5 and 10 m give equal or higher mean floors.

**Table 5:** Null distribution of the total bend *B* on straight matched phantoms (200 realizations per mast): mean, empirical *p*-value of the measured bend and its Holm-adjusted value for five masts; share of straight phantoms that a floor-based rule would classify as significant (*B* > 2*ε*_90_) or that exceed the floor (*B* > *ε*_90_), with the floors of Table 3; and false-positive rate (FPR) of the present test on 100 independent straight phantoms per mast (nominal 5%).

Mast	Bend (mm)	Null Mean (mm)	*p*	Holm *p*	*B* > 2*ε*_90_ (%)	*B* > *ε*_90_ (%)	Hold-Out FPR (%)
Foremast	160	50.8	<0.005	0.025	5.5	68.5	2
Mainmast	89	50.3	0.020	0.080	35.5	87.0	6
Middle Mast	45	49.9	0.61	1.00	21.5	84.5	7
Mizzenmast	36	50.4	0.79	1.00	19.5	83.0	4
Jiggermast	70	48.1	0.085	0.25	9.0	76.0	9

The measured UAV–TLS difference profiles constrain the model only loosely. Their autocorrelation falls to 1/e at a lag of 4.0–4.2 m (median over masts, transverse and longitudinal), close to the 3.9–5.6 m that the phantom error profiles show for every correlation length tested, because the 3 m kernel sets the short-range correlation of all profiles. The closest matches are the shortest and the longest correlation lengths (3.9–4.2 m for *l* ≤ 2.5 m and *l* = 40 m), so the statistic does not discriminate between them; their mean floors (16–21 mm and 18 mm) are lower than that of the default field, but the null threshold for *l* = 40 m is the highest of all models. Their RMS of 14–33 mm brackets the default mean floor. Recomputing the null distribution of Section 4.11 for each model shows how the verdicts depend on it (Table 4). The Foremast is significant under all fourteen models. The Mainmast is significant under eleven and not under the three models with the largest null thresholds (*l* = 40 m, the 60 mm amplitude, and the translation-only field). The Jiggermast is significant under seven models (under one of them at *p* = 0.0495, just below 0.05), and the Middle Mast and Mizzenmast only under the weakest field (15 mm), for which the null threshold is 26–28 mm.

### 4.8. Cross-Validation Against Terrestrial Laser Scanning

**Figure 13:**
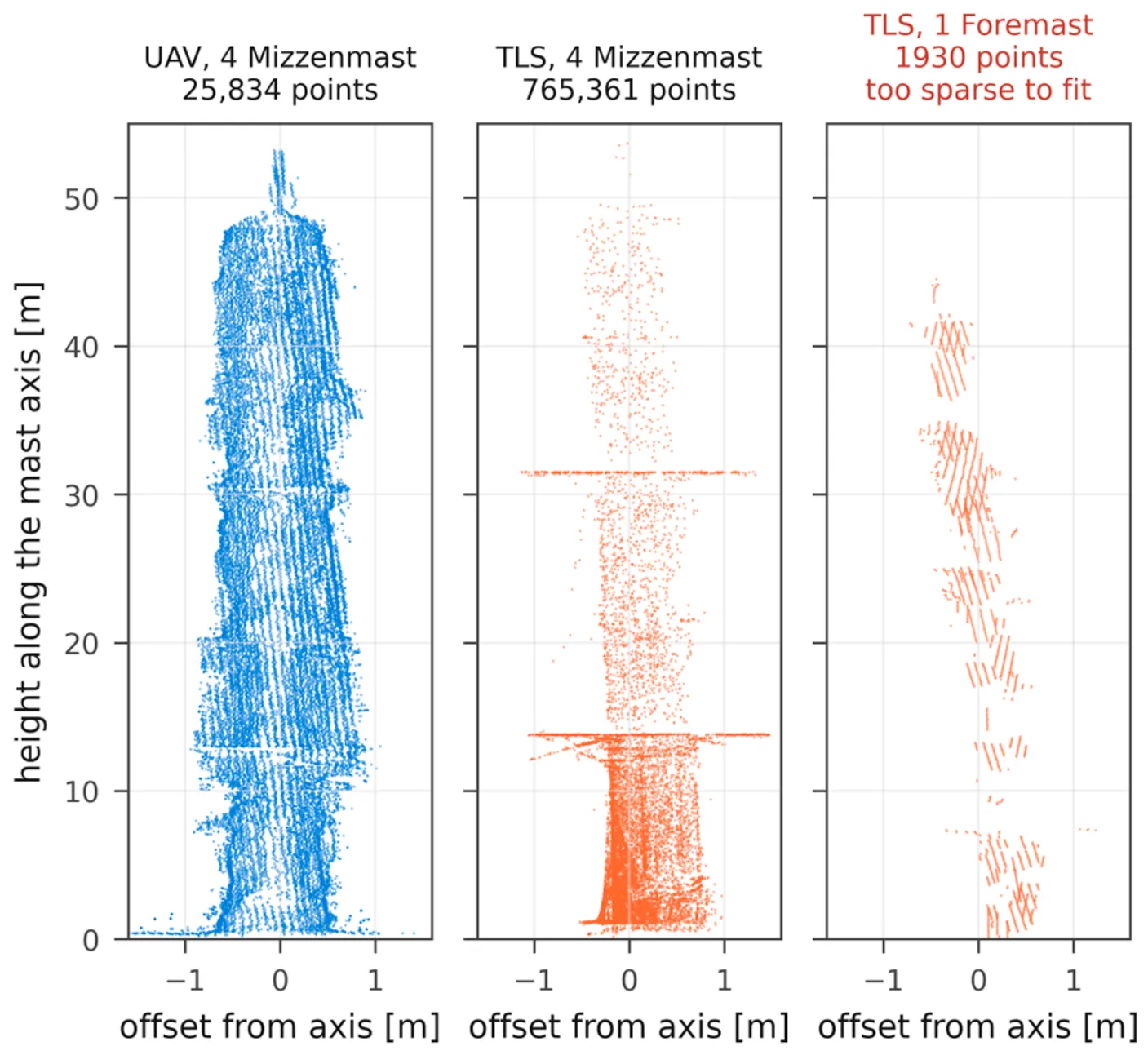
The same masts through two sensors, each shown in side view in its own axis frame (horizontal scale exaggerated): the UAV and TLS clouds of the Mizzenmast (the TLS cloud with its rigging returns) and the TLS cloud of the Foremast, which is too sparse to fit.

**Figure 14:**
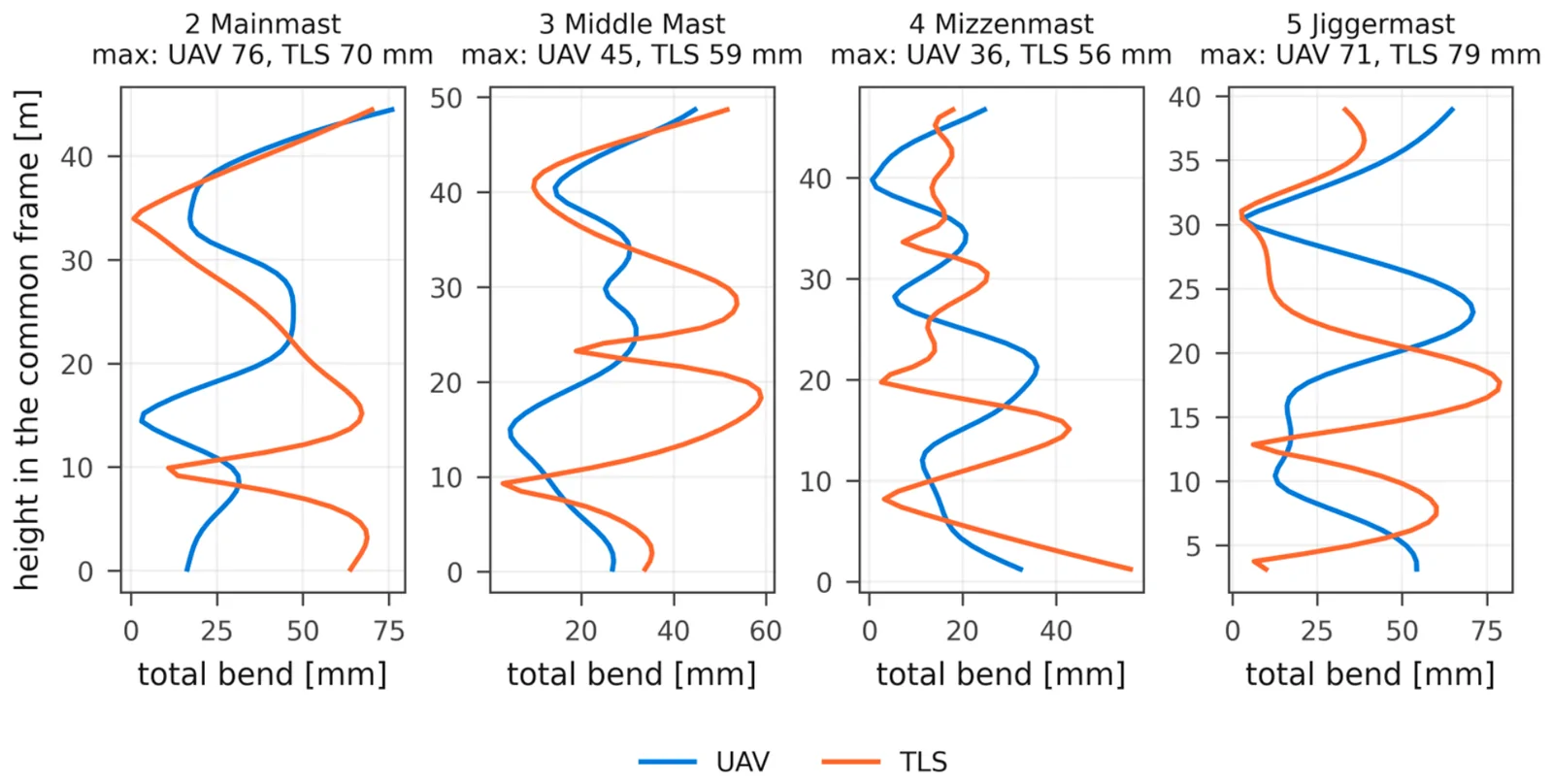
Total bending profiles from UAV photogrammetry and TLS for the four masts with adequate TLS coverage (Foremast excluded), processed by the identical pipeline (*L* = 3 m) and compared over the common height range after re-referencing to a common straight line.

**Figure 15:**
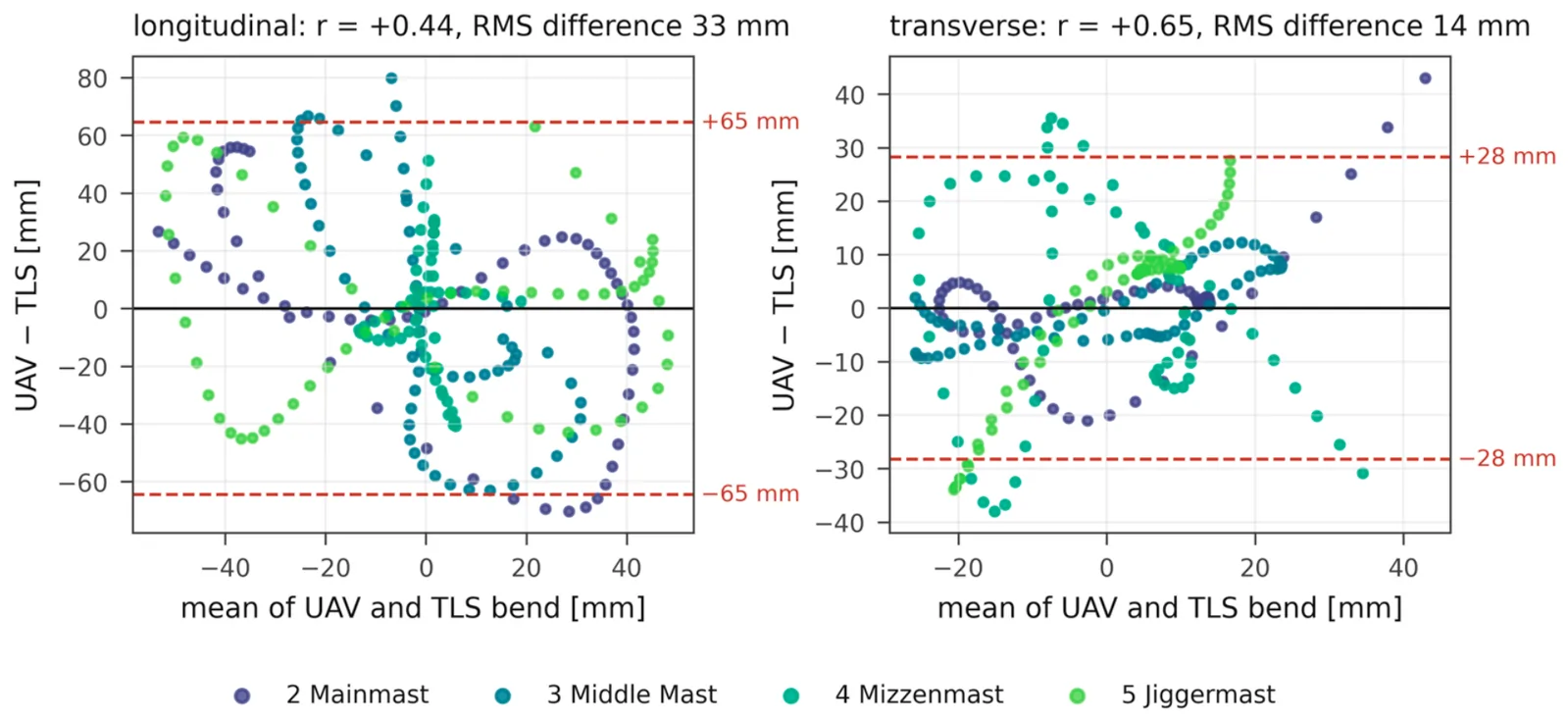
Bland–Altman comparison of UAV and TLS bends per height (four masts): longitudinal (**left**) and transverse (**right**) components with 95% limits of agreement; the bias is zero by construction (Section 4.8).

The most direct validation is a comparison with a second, independent sensor. The two sensors see the same masts very differently: the UAV yields a uniform but noisy SfM shell, whereas TLS yields a dense surface carrying rigging returns and, on the Foremast, coverage too sparse to fit (Figure 13). Over the four masts with adequate TLS coverage, the per-height Bland–Altman analysis (Section 3.11) gives RMS differences of 14 mm (transverse) and 33 mm (longitudinal), correlations of +0.65 and +0.44, and 95% limits of agreement of ±28 mm and ±65 mm (Figure 14 and Figure 15). The transverse differences stay within 30 mm at 92% of the heights. The longitudinal differences do so at 63% of the heights and reach 80 mm; on the Middle Mast and Mizzenmast, whose longitudinal bends (34 and 28 mm) are comparable to their error floors, the two longitudinal profiles are not positively correlated. The maximum bends over the common height range differ by 6–20 mm. These maxima are computed over the common height range after re-referencing and therefore differ from the full-span values of Table 1 (e.g., Mainmast, 76 against 89 mm).

The RMS differences between the two sensors (14–33 mm) are of the same order as the phantom-derived error floor (Section 4.5, Section 4.6 and Section 4.7), which the comparison therefore corroborates. The longitudinal component, although largely mast-specific (Section 4.4), agrees less well between the sensors than the transverse component. The limits of agreement change by at most 3 mm when the TLS heights are shifted by up to ±1 m per mast, so they are insensitive to residual vertical misregistration. The per-height differences are strongly autocorrelated (lag-1 coefficient ≈0.98), so that the approximately 240 per-height samples correspond to an effective sample size of only two to three per component, and four masts contribute. The mast-level block bootstrap gives 95% confidence intervals for the limits of agreement of ±18 to ±36 mm (transverse) and ±49 to ±77 mm (longitudinal). The limits of agreement therefore describe the observed profiles rather than precise population parameters; the conclusion drawn from them, agreement at the level of the error floor, holds across these intervals. Section 4.15 gives the comparison per mast, without re-referencing and with the TLS-side uncertainty. A limitation remains: the Foremast, which carries the largest UAV bend (160 mm), has TLS coverage too sparse to fit (1930 points), so its bend could not be confirmed independently.

A further observation concerns the orientation of the two datasets. After co-registration, the mast positions from the two sensors agree to within 0.1 m transversely and 0.7 m longitudinally, but the fitted axes differ in direction by 2.5–4.0°, and the TLS axes rake forward whereas the UAV axes rake aft, as designed. This is the expected consequence of the frame definition (Section 3.1): the TLS survey was acquired with the scanner’s inclinometer switched off [2], so its vertical axis is that of the registered scan set rather than gravity. A single rigid tilt of the TLS frame accounts for most of the discrepancy. Fitted to the four mast axes, it amounts to 3.3° (2.6° toward the bow and 2.0° transversely), which exceeds the design rake. After its removal, the axis directions of the two sensors agree to within 0.1–0.2° on the Mainmast and Middle Mast but still differ by 0.8° on the Mizzenmast and Jiggermast, about half the rake; the axis inclinations agree to within 0.15° on three masts and differ by 0.74° on the Mizzenmast, and the corrected TLS axis of the Jiggermast still does not rake aft. These residual differences may reflect changes in the rig between the two surveys, whose relative dates are unknown, or reconstruction error in either sensor; the absolute rakes in Table 1 should therefore be read with an uncertainty of up to about 0.8°. The tilt does not affect the bending estimates, because bending is measured relative to each mast’s own straight axis so that a rigid rotation of the point cloud cancels exactly. The tilt changes heights along each mast by less than 0.2%, which is covered by the ±1 m shift test above; it also moves each mast as a whole by up to 2.4 m in height, and pairing the heights after removing it changes the bending-profile differences by at most 7 mm (Section 4.15). Because the tilt is estimated from the masts themselves, it cannot be used to validate the absolute rake; the rake values in Table 1 are therefore derived from the UAV data alone.

### 4.9. Cross-Section Ovality

The median ovality is 8–27%, but a null test (the same second-harmonic estimator on a synthetically round ring with each slice’s own sampling and noise) yields only ≈2–3%. The excess, therefore, reflects real departures from roundness of the reconstructed sections rather than noise. We tested the hypothesis that it is caused by a one-sided welded mast track by masking the 30° sector with the largest mean radial residual before re-estimating ovality; masking reduced the median ovality only partly (e.g., Foremast 17.5 → 12.8%), and the excess over the null level remained at least threefold on every mast, so a single-sector attachment does not explain it. The cause is distributed, and ovality is reported as a limitation, not a deformation result. The TLS data suggest that most of it belongs to the reconstructed surface rather than to the steel tube: on the Mizzenmast and Jiggermast, which TLS covers over most of the circumference, the TLS ovality is only 2.5% and 5.5% (Section 4.14).

### 4.10. Inter-Mast Geometry and Absolute Dimensions

The mast positions give the inter-mast spacing directly: consecutive masts are 24.3, 27.5, 26.2, and 22.6 m apart (mean 25.1 m; Figure 4b). The TLS data give spacings that are 0.2–0.4 m (1–2%) longer, so the scale of the UAV reconstruction agrees with that of the TLS survey to within approximately 2%. The mast diameters, by contrast, differ by more than a scale error could explain. On the Mizzenmast and Jiggermast, which the TLS survey covers over most of the circumference (median 78% and 94% of 10° sectors; Section 4.15), the median UAV diameters exceed the TLS values by 13% and 8% (radius offsets of 59 and 36 mm); on the Mainmast and Middle Mast, where the TLS coverage is partial, the differences are larger (38% and 44%). The UAV reconstruction therefore places the mast surface outside the TLS surface. If this offset is uniform around the circumference, it does not move the slice centers, but the diameters in Table 1 describe the reconstructed surface rather than the steel tube. Section 4.14 shows that this offset, and the ovality of the reconstructed sections, move the slice centers by only a few millimeters.

### 4.11. Null Distribution of the Total Bend

The decision rule of Section 3.10 compares each measured total bend with its distribution for straight masts. For each mast, 200 matched phantoms with a perfectly straight centerline (zero bend, but the height, base radius, taper, point density, incoherent noise, scattered off-surface points, base flare, and correlated error of Section 4.6 and Section 4.7) were passed through the full pipeline (100 slices, *L* = 3 m), and their total bends *B* form the null distribution (Figure 8 and Table 5). Straight masts produce substantial apparent bends: the null mean of *B* is 48–51 mm, and its 95th percentile, the significance threshold, is 72–82 mm (Table 3). The null mean is 1.9–2.5 times the mean floor of the same mast, because the two measure different quantities. On an independent set of 100 straight phantoms per mast, the pointwise standard deviation of each bending component at a given height is 19–23 mm (median over heights), the RMS of the bending magnitude over height is 26–31 mm, and its maximum over height, the total bend, is 45–53 mm on average: the maximum of a correlated profile over a 42–54 m mast samples the upper tail of the pointwise distribution, and it combines two components.

Against these null distributions, the Foremast (*p* < 0.005) and the Mainmast (*p* = 0.020) are significant at the 5% level, whereas the Jiggermast (*p* = 0.085), the Middle Mast (*p* = 0.61), and the Mizzenmast (*p* = 0.79) are not resolved (Table 5). The *p*-values carry Monte Carlo uncertainty: for the Mainmast, 3 of the 200 null bends reached the measured value, and the 95% interval of the exceedance probability is 0.003–0.043; for the Jiggermast, 16 did, with an interval of 0.046–0.127. Adding the 100 independent phantoms changes the *p*-values only slightly (Mainmast 0.017, Jiggermast 0.096) and changes no verdict. Under the assumed error model, the test is calibrated: 5.6% of the independent straight phantoms exceeded the threshold of their mast (2–9% per mast), close to the nominal 5%. With five masts tested, a Holm correction leaves only the Foremast significant (adjusted *p* = 0.025; Mainmast 0.080).

A rule based on the error floor instead, which declares a bend significant if it exceeds twice the 90th percentile of the floor of its mast, is not calibrated. With the floors of Table 3, it would classify 18% of the straight phantoms as significantly bent (6–36% per mast, highest for the Mainmast, whose floor is lowest) and 63% against twice the mean floor; 80% of the straight phantoms exceed the 90th percentile of the floor itself (*B* > *ε*_90_). An intermediate class between *ε*_90_ and 2*ε*_90_ is therefore not meaningful, and the bends of the Middle Mast, Mizzenmast, and Jiggermast are reported as not resolved. The null distribution also depends on the assumed error model, though not in the same way as the floor: recomputed for each of the fourteen models of Table 4, its 95th percentile ranges from 26–28 mm for the weakest field to 108–150 mm for the longest correlation length, which lowers the floor but raises the null threshold most, and the verdicts change accordingly (Section 4.7). The verdicts above rest on the default model, whose correlated amplitude equals the whole measured per-slice scatter (Section 4.5).

### 4.12. Response of the Smoother and Component Ablations

**Figure 16:**
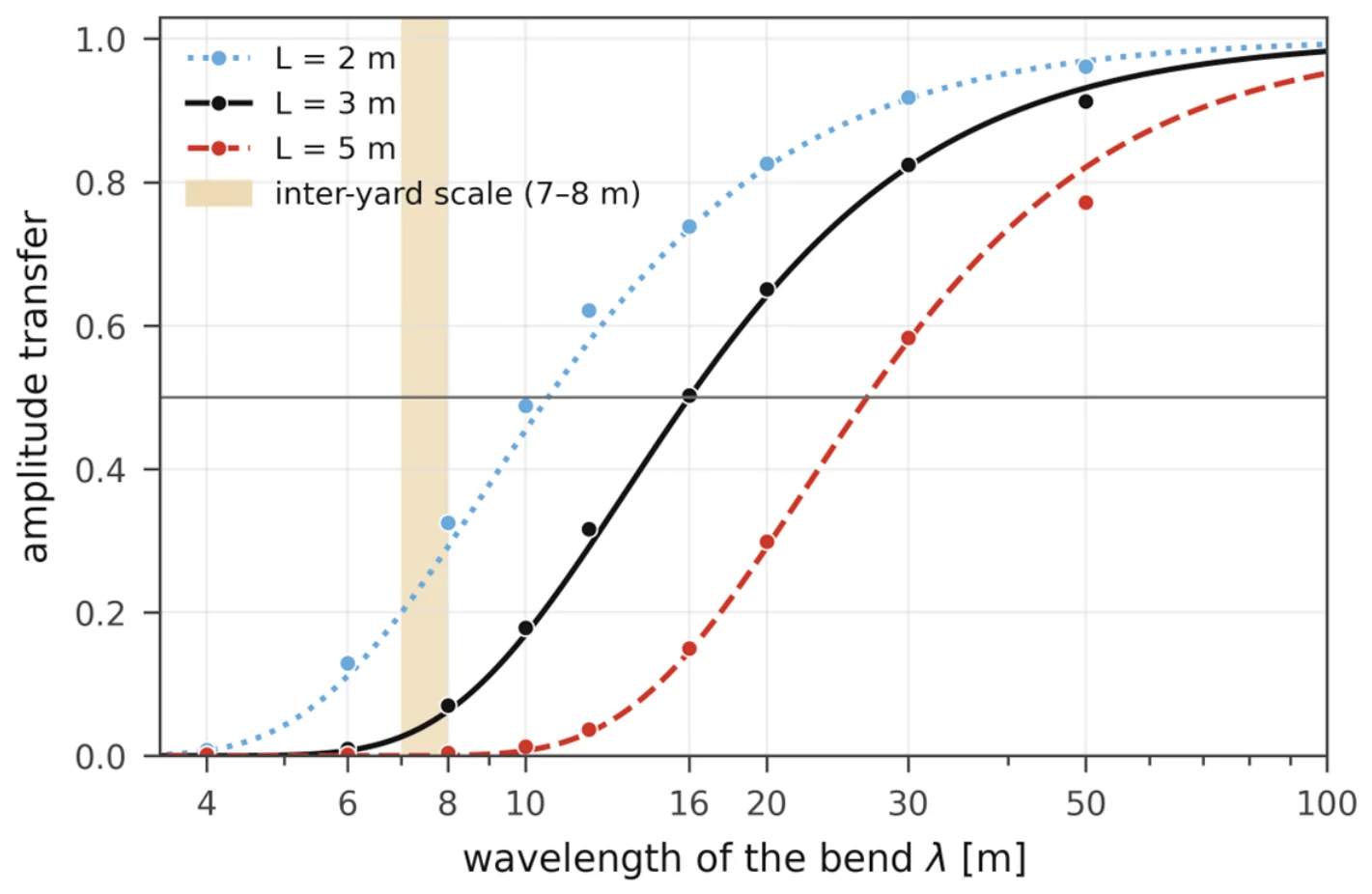
Amplitude transfer of the kernel smoother for sinusoidal bends of wavelength *λ*: theory, *H*(*λ*) = exp(−2π^2^*L*^2^/*λ*^2^) (lines), and the full pipeline on phantoms (dots), for *L* = 2, 3, and 5 m. The shaded band marks the inter-yard scale (7–8 m); the horizontal line marks 50% transfer, reached at *λ* = 16 m for *L* = 3 m.

Figure 16 separates noise suppression from attenuation of genuine bending. Phantoms with a sinusoidal bend of known wavelength *λ* (4–50 m; amplitude 50 mm; 50 m mast; 5 mm noise; three realizations each) were processed with *L* = 2, 3, and 5 m, and the recovered amplitude was compared with the true one away from the ends of the mast. The full pipeline follows the transfer function *H*(*λ*) of Section 3.7 to within 0.05 at all wavelengths tested; the largest departures occur at 50 m, which is comparable to the mast height and where the straight reference line absorbs part of the wave. With *L* = 3 m, bending at the inter-yard scale (*λ* ≈ 7–8 m) is attenuated to at most 7% of its amplitude, so it is not resolved; the transfer is 18% at 10 m, 50% at 16 m, 65% at 20 m, and 82% at 30 m. The decrease of the bend with increasing *L* (Section 4.2) therefore combines noise suppression with attenuation of genuine bending at wavelengths shorter than about 30 m. The measured profiles are dominated by long wavelengths: with *L* = 1 m, 83–95% of their bending power lies at wavelengths of 16 m or longer on four masts and 61% on the Mizzenmast, the mast with the smallest bend. The reported bends thus describe deformation over roughly a third of the mast height or more; a localized deformation, such as a kink between two yards, would require a smaller *L* and, with it, a higher error floor.

Table 6 isolates the components of the final pipeline (100 slices, *L* = 3 m) by disabling each separately: base-flare removal, rigging-band suppression, covariance weighting (equal weights in the smoother), and axis refinement (slices cut along the PCA seed). On the real masts, no single ablation changes a bend by more than 9.3 mm: covariance weighting has the largest effect (Middle Mast 45 → 36 mm, Mainmast 89 → 94 mm), axis refinement changes the bends by at most 3.8 mm, flare removal by at most 2.2 mm, and band suppression by at most 1.8 mm. On the matched phantoms, flare removal is the only component whose removal raises the floor appreciably (mean 21.9 → 25.5 mm, 90th percentile 34.7 → 44.1 mm); the others change it by at most 0.2 mm (mean) and 1.2 mm (90th percentile). The three slice-level components overlap, because flared slices have large center covariances and are non-circular, so the weighting and the band test also act on them. Disabling any one or two of them changes the measured bends by at most 9.3 mm, whereas disabling all three raises the Mizzenmast bend from 36 to 114 mm, the flare failure of the unsmoothed baseline (Section 4.2) in another form. On the phantoms, the combinations do not add simply, and disabling all three leaves the floor almost unchanged (22.2 mm), because the phantom flare is axisymmetric: its slices are wider than the tube but centered on its axis. The phantom therefore understates the value of these safeguards against the asymmetric deck fittings of the real masts.

### 4.13. Kernel Bandwidth and Stability of the Verdicts

The bends decrease with the bandwidth (Section 4.2), as the transfer function predicts (Section 4.12). Because the error floor and the null distribution also depend on *L*, both were recomputed for *L* = 2, 3, 4, and 5 m with the same phantoms and seeds (Table 7). The floors fall with *L* (means of 21–30 mm for *L* = 2 m and 17–20 mm for *L* = 5 m), and so do the null thresholds (81–89 mm and 58–67 mm). The verdicts do not change: the Foremast (*p* < 0.005) and the Mainmast (*p* = 0.015–0.040) are significant at the 5% level for every *L;* the Middle Mast, Mizzenmast, and Jiggermast are not (*p* > 0.06); and after a Holm correction for five masts, only the Foremast remains significant. *L* = 3 m was fixed before the analysis from two considerations that do not involve the bends: the kernel must bridge the gaps left by the removed band and flare slices, which reach 1.3–2.2 m on the five masts, and it should describe the bending of the mast as a whole, at wavelengths of a third of the mast height or more (*λ*_1/2_ = 16 m), rather than features at the 7–8 m spacing of the yards, such as residual rig-band effects. Because the null distribution is recomputed for the bandwidth used, the test holds its level at any fixed *L*, and the stability of the verdicts over *L* = 2–5 m shows that they do not depend on this choice.

### 4.14. Circle Versus Ellipse Centers

The centerline is built from circle-fit centers, whereas the UAV surface lies outside the TLS surface (Section 4.10) and its sections are oval (Section 4.9). A uniform radial offset is absorbed by the fitted radius and does not move the center, and a symmetric, second-harmonic distortion such as ovality moves it only where the ring is incompletely covered; the UAV slices cover a median of 100% of the circumference (10th percentile 92%; Table 2). We tested both points. On the real masts, replacing each circle center by the center of a direct least-squares ellipse fit [36] to the same inliers changes the bends by at most 3.8 mm (Jiggermast 70 → 66 mm, the others by at most 2.6 mm), and the two centerlines differ by 1.6–5.9 mm RMS, well below the floors; no verdict changes. On phantoms with a 100 mm bend and 20 mm noise, a uniform offset of 50 mm, an ovality of 10% with a fixed orientation or of 25% with an orientation turning along the height, and 60° gaps in 30% of 1 m bands leave the bending-profile RMSE at 3.7–4.0 mm with either model, against 3.9 mm for round sections.

The TLS data suggest that the ovality belongs largely to the reconstruction: on the Mizzenmast and Jiggermast, which TLS covers over most of the circumference, the median TLS ovality is 2.5% and 5.5% (round-ring null 0.3%), against 19% and 27% from the UAV data. On the Mainmast and Middle Mast, TLS gives 27% and 29% (null 10% and 5%), but there it sees only about half of the circumference, together with rigging returns, so the attribution rests on the two masts with the most complete coverage. What neither section model can detect is a one-sided radial bias of the reconstructed surface that varies along the height, for example, a viewing-dependent offset: an S-shaped bias of 20 mm raises the phantom RMSE from 3.9 to 8.1 mm with both models. Such a bias is bounded only by the comparison with TLS (Section 4.8 and Section 4.15).

### 4.15. UAV–TLS Comparison per Mast and Without Re-Referencing

Table 8 repeats the comparison of Section 4.8 per mast and with progressively less adjustment. In the common ship frame as co-registered, the two centerlines differ by 0.4–0.8 m RMS, mainly because of the 3.3° tilt of the TLS frame. After one rigid alignment for all masts, they differ by 46–53 mm transversely and by 134–374 mm longitudinally, and after a further constant offset per mast by 43–53 mm and 39–105 mm. These residuals contain differences of mast position along the ship (the inter-mast spacings differ by 0.2–0.4 m; Section 4.10) and of axis direction (up to 0.8° after the common tilt is removed; Section 4.8), which the bending estimand excludes by definition, because bending is measured against each mast’s own straight axis; whether they reflect a change of the rig between the two surveys or a residual registration error cannot be decided without the date of the UAV survey. With a straight line removed per mast and the heights paired as in Section 4.8, the differences are 7–20 mm transversely and 21–42 mm longitudinally. Pairing the heights after rotating the TLS clouds by the common tilt, which moves the individual masts by up to 2.4 m in height, changes these differences by at most 7 mm (Mainmast 15 and 28 mm, Middle Mast 6 and 43 mm, Mizzenmast 20 and 21 mm, and Jiggermast 17 and 30 mm) and the pooled RMS differences only from 14 and 33 mm to 15 and 31 mm.

The TLS side contributes little to these differences where its coverage is most complete but more where it is not. The per-slice center uncertainty of the TLS fits is 1.0–8.4 mm, and TLS-like phantoms with 3 mm noise, the measured point density, rigging returns, and the measured one-sided angular coverage give a bending-profile RMSE of 4–6 mm for round sections. Partial coverage, however, makes the TLS centers sensitive to ovality: with the 2.5–5.5% ovality that TLS measures on the Mizzenmast and Jiggermast, the RMSE rises to 7–15 mm on the Mainmast and Middle Mast, which TLS covers over only about half of the circumference. If the UAV and TLS errors are independent, the expected RMS difference is therefore 21–25 mm with the mean floors of Table 3 and 29–35 mm with their 90th percentiles, depending on the mast and on the TLS ovality. The transverse differences (7–20 mm) are smaller than this, and the longitudinal differences on the Mizzenmast and Jiggermast (21 and 31 mm) lie within it. The longitudinal difference on the Middle Mast (42 mm) exceeds the 90th-percentile expectation for that mast (32–34 mm), and that on the Mainmast does so with the heights paired as in Section 4.8 (35 against 29–33 mm) but not after the rotation (28 mm); the longitudinal profiles of the Middle Mast and Mizzenmast are not positively correlated (Section 4.8). The comparison therefore corroborates the magnitude of the UAV floor, except in the longitudinal direction on the two masts with half TLS coverage, where the excess cannot be assigned to either sensor.

## 5. Discussion

*What is established.* The pipeline recovers the rake and centerline bending of each mast, with per-mast error floors of 20–27 mm on average and 29–40 mm at the 90th percentile, whose magnitude is corroborated by an independent sensor, except longitudinally on the two masts that TLS covers over only about half of the circumference (Section 4.15). Tested against the null distribution of the total bend of straight masts, bending is significant at the 5% level for Foremast and Mainmast and is not resolved for Middle Mast, Mizzenmast, and Jiggermast. The Foremast verdict holds under all fourteen error models of Table 4 and, under the default model, after a Holm correction for five masts; the Mainmast verdict holds under eleven of the models but not after the correction, and the Jiggermast, not resolved under the default model, is significant under seven. The largest bend, on the Foremast, is not confirmed by the TLS data because of insufficient coverage, and its longitudinal component also dominates the candidate shared mode, so the two significant results are supported differently: the Foremast statistically, the Mainmast only in part, through the agreement with TLS of its transverse bending profile over the common height range, which does not extend to its longitudinal component or to its full-span bend. The Foremast verdict is thus the only one that holds under every error model, but it rests on the UAV data alone. The bending profiles from the two sensors agree with RMS differences of 14–33 mm. The verdicts are unchanged for kernel bandwidths of 2–5 m, with the floor and the null distribution recomputed for each bandwidth (Section 4.13), and replacing the circle centers by ellipse centers changes the bends by at most 3.8 mm (Section 4.14).

*What is not established.* A single static capture cannot separate a permanent (plastic) set from elastic deflection under rigging, wind, or thermal load, so results describe the as-built centerline at capture time. Cross-section ovality exceeds the round-ring null level, but where TLS covers most of the circumference it measures only 2.5–5.5%, so most of the UAV ovality appears to belong to the reconstructed surface; ovality is therefore not reported as a deformation result. The absolute scale of the UAV reconstruction could be checked only against the TLS survey, with which it agrees to within approximately 2% (Section 4.10). Its effect on the bending conclusions is bounded, because scale error is multiplicative, whereas bending is a differential quantity: a fractional scale error δ changes a 160 mm bend by only 160δ mm (3.2 mm for δ = 2%), which is negligible against the error floor. The UAV diameters exceed the TLS diameters by 8–13% where TLS covers most of the circumference, so the diameters describe the reconstructed surface rather than the steel tube.

*Acquisition metadata.* The contracting surveyor did not supply the acquisition and processing metadata of the UAV survey (Section 3.1). For a metrology study, this is a serious limitation: without the camera model, ground sample distance, image overlap, ground-control distribution, and checkpoint residuals, the precision of the reconstruction cannot be predicted from the survey design, and the error floor cannot be attributed to a specific cause. The accuracy statements of this paper therefore rest entirely on a posteriori evidence that does not depend on the metadata: phantoms matched to the measured cloud statistics, with the dependence of the floor and of the verdicts on the assumed error model quantified (Table 4); the internal shared-curvature check; and the independent TLS survey. Photogrammetric doming, which depends on the image geometry, can be assessed only indirectly, through the shared-curvature check and the TLS comparison. The absolute scale could therefore be checked only against the TLS survey (Section 4.10); no provably straight scene feature was available for a straightness check, and the date of the UAV survey relative to the TLS survey is unknown. The berth condition bounds but does not eliminate the elastic contribution to the measured deflection. In practice, point clouds are often delivered without processing reports, and the a posteriori validation used here does not require them. For future campaigns, the acquisition metadata should be recorded.

*Threats to validity.* The deck-collar flare (taper-consistency test; a large effect in an unsmoothed baseline, which in the final pipeline the weighting and the band test also control), the periodic attachment bands (spike detection), and the candidate reconstruction-wide curvature (five-mast shared-mode test, inconclusive on these data) are identified; the first two are bounded quantitatively, and the third only through the TLS agreement and the synthetic doming recovery. The significance verdicts rest on a null distribution computed under an assumed correlated-error model, and Table 4 shows which of them depend on it; the bends describe deformation at wavelengths of about 16 m and longer (Section 4.12). A one-sided bias of the reconstructed surface that varies along the height would move the slice centers whatever section model is used, and only the TLS comparison bounds it (Section 4.14 and Section 4.15).

*Generality.* The same deterministic sequence is expected to extend to other tall, tapered, occluded cylindrical structures imaged by UAV (wind-turbine towers, chimneys, and flare stacks), particularly where several similar structures are captured together so the shared-mode artifact test applies. We state this as an untested extension.

## 6. Conclusions

This paper has presented a deterministic, self-referential method for measuring the bending of large, occluded, tapered masts directly from a UAV photogrammetric point cloud, replacing a manual laser-scanning and CAD-based reconstruction that depended on a fragile registration to a design model [2]. Each mast is treated as a tapered generalized cylinder: a principal-component axis seed is refined by deterministic iteratively reweighted least squares, the point cloud is divided into robustly circle-fitted slices along the refined axis, the deck-collar flare and rigging-attachment bands are removed by data-driven tests, and an uncertainty-weighted centerline is decomposed into a fitted straight axis, which gives the rake, and a bending residual. Deformation is thus measured relative to each mast’s own straight axis, without a CAD model, and repeated runs on identical input give identical results.

Applied to the five masts of the *Golden Horizon*, the method recovers an axis rake of 1.2–1.9°, consistent with the intended aft rake of approximately 2°, and kernel-smoothed bending maxima of 36–160 mm at wavelengths of about 16 m and longer. Three results carry the methodological weight. First, the deck-collar flare must be controlled: in an unsmoothed baseline variant of the pipeline, omitting its removal increases the estimated bending of one mast from 87 to 410 mm, and in the final pipeline, in which the flare test, the covariance weighting, and the band test overlap, disabling all three raises the bend of the same mast from 36 to 114 mm. Second, the achievable accuracy is governed by spatially correlated reconstruction error rather than by independent per-point noise; a white-noise error model overstates the accuracy by a factor of about three under the default, deliberately pessimistic correlated amplitude (7 mm against 20–27 mm on matched phantoms), and the correlated error is largely invisible in the per-slice fitting residual. Third, an independent TLS survey processed by the identical pipeline gives bending profiles that differ from the UAV profiles by 14–33 mm RMS (95% limits of agreement ±28–65 mm), consistent with the synthetically derived error floor, except longitudinally on the two masts that TLS covers over only about half of the circumference. Tested against the null distribution of the total bend of straight synthetic masts, which a profile-wide error floor cannot replace, bending is significant at the 5% level for two masts (Foremast and Mainmast) and not resolved for three (Middle Mast, Mizzenmast, and Jiggermast); after correction for testing five masts, only the Foremast bend remains significant. The verdicts also depend on the assumed error model (Table 4): the Foremast is significant under all fourteen models examined, the Mainmast under eleven, the Jiggermast under seven, and the Middle Mast and Mizzenmast only under the weakest one (15 mm amplitude), so only the Foremast verdict does not depend on this assumption. The largest bend, on the Foremast, could not be confirmed independently because its TLS coverage was insufficient. With five masts, the shared-curvature test for doming is inconclusive. The Mainmast bend, although statistically weaker, is the one that could be compared with TLS: the two sensors agree closely in its transverse component over the common height range but less closely in its longitudinal component. Neither significant bend is therefore fully confirmed by an independent sensor.

The study also delimits what the data can support. Cross-section ovality exceeds the round-ring null level, and the TLS data suggest that most of the excess belongs to the reconstructed surface rather than to the steel tube; it is therefore reported as a limitation rather than a deformation result. A single static acquisition cannot separate permanent set from elastic deflection under rigging, wind, or thermal load, so the results describe the as-built centerline at the time of capture. The absolute scale could be checked only against the TLS survey, with which it agrees to within approximately 2%, and its effect on the bending estimates is small; the UAV diameters, by contrast, exceed the TLS diameters and describe the reconstructed surface rather than the steel tube. Future work should record the acquisition metadata of the UAV survey, incorporate a stochastic model of the point coordinates into the slice fit, apply a repeated-acquisition or differential-load protocol to separate permanent from elastic deformation, and use higher-fidelity capture for ovality. Because no component of the pipeline is specific to masts, the method is expected to transfer to other tall, tapered, occluded cylindrical structures imaged by UAV, such as wind-turbine towers, chimneys, and flare stacks.

## Figures and Tables

**Table 1:** Per-mast results (kernel-smoothed, *L* = 3 m; rake removed). Transverse and longitudinal bends are the maxima of the absolute component profiles; the total bend is the maximum of the resultant lateral deviation. Median diam. is the median fitted diameter of the reconstructed UAV surface over the retained slices (see Section 4.10). *σ*_stat_ is the median random uncertainty of a slice center and is not the limiting uncertainty (Section 4.5, Section 4.6 and Section 4.7).

Mast	Points	Rake (°)	Median Diam. (mm)	Transverse Bend (mm)	Longitudinal Bend (mm)	Total Bend (mm)	*σ*_stat_ (mm)
Foremast	14,752	1.38	678	61	148	160	6.4
Mainmast	18,069	1.86	704	82	48	89	6.1
Middle Mast	17,152	1.73	749	30	34	45	5.2
Mizzenmast	25,834	1.45	1030	35	28	36	8.2
Jiggermast	17,586	1.20	959	37	60	70	11.3

**Table 2:** Per-slice statistics governing fit accuracy: real UAV clouds (pooled over five masts) against the phantom before and after matching.

Statistic	Real	Phantom (Original)	Phantom (Matched)
Radial residual *σ*, median (mm)	45.6	9.0	46.2
Radial residual *σ*, 90th pct (mm)	112.2	11.2	75.4
Points trimmed as outliers (%)	5.6	0.2	6.6
Angular coverage, 10th pct (%)	91.7	100.0	100.0
Point density (points/m)	387	391	423

**Table 3:** Per-mast error floor from geometry- and noise-matched phantoms under correlated error (mean and 90th percentile of the bending-profile RMSE over 10 realizations), 95th percentile of the null distribution of the total bend (200 straight phantoms; Section 4.11), and the resulting verdict at the 5% level.

Mast	Height (m)	Taper	Bend (mm)	Floor: Mean (mm)	Floor: p90 (mm)	Null: p95 (mm)	Verdict (5%)
Foremast	51.3	0.0035	160	27.1	40.1	81.1	significant
Mainmast	52.7	0.0049	89	20.1	29.0	80.4	significant
Middle Mast	53.7	0.0049	45	21.1	31.7	77.0	not resolved
Mizzenmast	53.2	0.0008	36	21.0	33.2	82.2	not resolved
Jiggermast	42.1	0.0030	70	20.0	34.7	72.1	not resolved

**Table 4:** Sensitivity of the error floor and of the verdicts to the correlated-error model (matched phantoms): mean and 90th percentile of the bending-profile RMSE (10 realizations per mast, 50 per model); range over the five masts of the 95th percentile of the null distribution of the total bend (100 straight phantoms per mast; default model, 200, Table 5); and the verdict of each mast at the 5% level, bow to stern (S, significant; N, not resolved). GP: Gaussian-process field with correlation length *l* and a root mean square (RMS) of 45 mm per component; anisotropy is the RMS ratio in the plane of the bend to the perpendicular direction.

Error Model	Floor: Mean (mm)	Floor: p90 (mm)	Null p95 (mm)	Verdicts, Masts 1–5
Default field, 45 mm (Table 3 and Table 5)	21.9	34.7	72–82	S S N N N
GP, *l* = 1 m	16.4	21.6	49–56	S S N N S
GP, *l* = 2.5 m	21.1	27.7	60–68	S S N N S
GP, *l* = 5 m	23.0	30.6	70–78	S S N N N
GP, *l* = 10 m	22.1	31.6	67–77	S S N N S
GP, *l* = 20 m	16.7	27.5	63–74	S S N N S
GP, *l* = 40 m	18.2	34.6	108–150	S N N N N
Default field, 15 mm	10.4	14.8	26–28	S S S S S
Default field, 30 mm	16.3	25.3	51–56	S S N N S
Default field, 60 mm	28.6	47.5	96–113	S N N N N
GP, *l* = 10 m, anisotropy 1:2	17.1	26.2	70–82	S S N N N
GP, *l* = 10 m, anisotropy 2:1	26.3	37.6	73–83	S S N N N
GP, *l* = 10 m, translation only	28.7	39.9	90–103	S N N N N
GP, *l* = 10 m, 80% angular	14.3	22.7	42–53	S S N N S

**Table 6:** Ablations under the final configuration (100 slices per mast, *L* = 3 m): total bend of each real mast (mm; masts 1–5 from bow to stern: Foremast, Mainmast, Middle Mast, Mizzenmast, and Jiggermast) and error floor on the matched phantoms (mean and 90th percentile of the bending-profile RMSE over 50 realizations, all masts pooled).

Configuration	Mast 1	Mast 2	Mast 3	Mast 4	Mast 5	Floor: Mean (mm)	Floor: p90 (mm)
Full pipeline	159.6	89.4	45.4	36.0	70.2	21.9	34.7
No base-flare removal	159.6	89.4	45.4	36.0	72.4	25.5	44.1
No rigging-band suppression	159.2	88.8	47.2	35.9	70.3	21.7	33.5
No covariance weighting	157.3	94.3	36.1	38.3	74.4	22.0	34.7
No axis refinement	157.0	88.4	49.2	36.5	70.2	21.8	34.5
No flare removal or band suppression	159.2	89.3	48.0	38.2	72.5	25.1	43.0
No flare removal or weighting	157.3	94.3	36.1	38.3	77.9	24.1	40.1
No band suppression or weighting	153.3	93.9	38.6	41.2	74.9	21.0	32.7
None of the three	153.3	93.2	51.7	113.7	79.4	22.2	35.4

**Table 7:** Bends, error floors, null distributions, and verdicts for each kernel bandwidth *L*, all recomputed with that *L* (10 matched and 200 straight phantoms per mast): *λ*_1/2_, wavelength transferred with half of its amplitude; floor (mean) and null threshold (95th percentile) as ranges over the five masts; *p*-values of the two masts closest to the threshold (Foremast *p* < 0.005, Middle Mast and Mizzenmast *p* > 0.5 for every *L*); verdicts at the 5% level, bow to stern (S, significant; N, not resolved).

*L* (m)	*λ*_1/2_ (m)	Bends, Masts 1–5 (mm)	Floor: Mean (mm)	Null: p95 (mm)	*p*, Mainmast	*p*, Jiggermast	Verdicts
2	10.7	176, 99, 46, 48, 81	21–30	81–89	0.015	0.070	S S N N N
3	16.0	160, 89, 45, 36, 70	20–27	72–82	0.020	0.085	S S N N N
4	21.3	147, 77, 42, 30, 57	18–24	65–74	0.030	0.17	S S N N N
5	26.7	136, 67, 37, 23, 49	17–20	58–67	0.040	0.19	S S N N N

**Table 8:** UAV–TLS comparison per mast (transverse/longitudinal): RMS difference of the centerlines in the common ship frame as co-registered, after one rigid alignment for all masts (a rotation removing the common tilt and a horizontal translation), after a further constant offset per mast, and after a straight line per mast (the bending estimand, heights paired as in Section 4.8, without the rotation); correlation of the bending profiles; median angular coverage of the TLS slices (share of 10° sectors containing points); and bending-profile RMSE of TLS-like phantoms with that coverage, for round sections and with the 2.5–5.5% ovality measured by TLS on the Mizzenmast and Jiggermast.

Mast	Co-Registered (mm)	Global Alignment (mm)	Plus Offset per Mast (mm)	Line Removed (mm)	Correlation	TLS Coverage (%)	TLS Phantom (mm)
Mainmast	498/805	49/374	45/94	11/35	0.84/0.63	50	4–15
Middle Mast	537/732	49/149	49/105	7/42	0.96/−0.36	58	4–13
Mizzenmast	511/598	53/134	53/68	20/21	0.37/−0.51	78	4–9
Jiggermast	405/538	46/354	43/39	16/31	0.82/0.68	94	6

## Data Availability

The TLS point clouds of the *Golden Horizon* masts were provided by Brodosplit, and the UAV point clouds were provided by an external surveyor; both are available from the corresponding author on reasonable request, subject to permission from the data owners. The code and the scripts that reproduce all results are openly available at https://github.com/akskeli/mastfit/.

## Abbreviations

The following abbreviations are used in this manuscript:

3D	three-dimensional
CAD	computer-aided design
FPR	false-positive rate
GP	Gaussian process
IRLS	iteratively reweighted least squares
LiDAR	light detection and ranging
MAD	median absolute deviation
PCA	principal component analysis
RANSAC	random sample consensus
RGB-D	red–green–blue–depth
RMS	root mean square
RMSE	root-mean-square error
SfM	structure from motion
TLS	terrestrial laser scanning
UAV	unmanned aerial vehicle
